# Machine Learning-Boosted Docking Enables the Efficient Structure-Based Virtual Screening of Giga-Scale Enumerated Chemical Libraries

**DOI:** 10.1021/acs.jcim.3c01239

**Published:** 2023-09-01

**Authors:** Toni Sivula, Laxman Yetukuri, Tuomo Kalliokoski, Heikki Käsnänen, Antti Poso, Ina Pöhner

**Affiliations:** † School of Pharmacy, University of Eastern Finland, Kuopio FI-70211, Finland; ‡ CSC—IT Center for Science Ltd., Espoo FI-02101, Finland; § Computational Medicine Design, Orion Pharma, Orionintie 1A, Espoo FI-02101, Finland; ∥ Department of Pharmaceutical and Medicinal Chemistry, Institute of Pharmaceutical Sciences, Eberhard Karls University, Tübingen DE-72076, Germany; ⊥ Cluster of Excellence iFIT (EXC 2180) “Image-Guided and Functionally Instructed Tumor Therapies”, University of Tübingen, Tübingen DE-72076, Germany; ¶ Tübingen Center for Academic Drug Discovery & Development (TüCAD2), Tübingen DE-72076, Germany

## Abstract

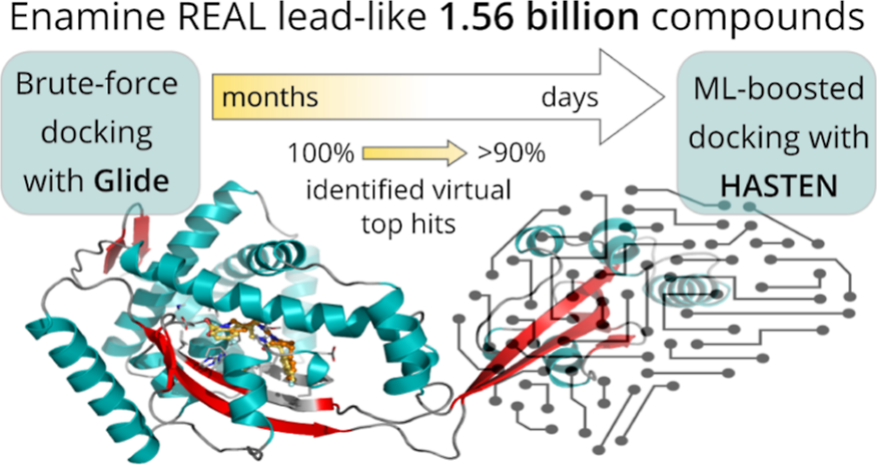

The emergence of ultra-large screening libraries, filled to the brim with billions of readily available compounds, poses a growing challenge for docking-based virtual screening. Machine learning (ML)-boosted strategies like the tool HASTEN combine rapid ML prediction with the brute-force docking of small fractions of such libraries to increase screening throughput and take on giga-scale libraries. In our case study of an anti-bacterial chaperone and an anti-viral kinase, we first generated a brute-force docking baseline for 1.56 billion compounds in the Enamine REAL lead-like library with the fast Glide high-throughput virtual screening protocol. With HASTEN, we observed robust recall of 90% of the true 1000 top-scoring virtual hits in both targets when docking only 1% of the entire library. This reduction of the required docking experiments by 99% significantly shortens the screening time. In the kinase target, the employment of a hydrogen bonding constraint resulted in a major proportion of unsuccessful docking attempts and hampered ML predictions. We demonstrate the optimization potential in the treatment of failed compounds when performing ML-boosted screening and benchmark and showcase HASTEN as a fast and robust tool in a growing arsenal of approaches to unlock the chemical space covered by giga-scale screening libraries for everyday drug discovery campaigns.

## Introduction

Virtual screening (VS) approaches utilizing molecular docking are a common choice in the early stages of structure-based drug discovery projects. Typically, their objective is to find the initial small molecule hits predicted to bind to a previously unexplored target, or to discover novel scaffolds in an unbiased way when confirmed binders of the studied target are already known. Especially in the initial screening steps, VS often utilizes large and diverse screening libraries to pool the most promising candidates from a large chemical space.^1−3^ Chemical libraries of off-the-shelf or readily synthesizable (make-on-demand) compounds are popular to ensure that the docking predictions can be validated in biochemical assays in a timely manner without the need to factor in potentially time-consuming organic synthesis. In recent years, such libraries have continuously grown and today often cover a vast chemical space with compound numbers on the billion scale.^4^ For example, at the beginning of this project, the Enamine REAL library of lead-like compounds had a size of 1.56 billion (March 2021), whereas the current version has 3.93 billion compounds (August 2023).^5^ What is a huge leap forward in terms of access to diverse chemical space and straightforward validation of docking-based hypotheses, at the same time becomes a challenge for docking-based VS: since conventional brute-force docking visits every compound in a chemical library, screening billions of compounds is often no longer feasible, both in terms of the required time and the computational power.

On the other hand, structure-based VS of such libraries has produced hits of exceptional quality for several targets (see, e.g., studies by Lyu et al.,^2^ Stein et al.,^3^ and Kaplan et al.^6^). One key study to motivate the work with such large libraries was presented by Lyu et al.^2^ Their docking of around 100 million compounds to two targets, β-lactamase AmpC, and D_4_ dopamine receptor, did not only result in the discovery of novel ligands for both targets, but they made critical observations for many studies to follow: first, they found that if docking enriches true positives over decoys on the small scale, enrichment can be expected to be translated to the ultra-large scale. Second, they demonstrated that, at least for the D_4_ receptor, the novel ligands could not have been found by, for example, docking only cluster representatives instead of the full library. Consequently, as compound libraries grow even bigger, there is a clear need for faster and more efficient methods to screen entire ultra-large libraries.

There are various approaches to tackle the computational expense associated with billion-scale structure-based VS. One strategy is to grow compounds from fragments instead of docking full-size molecules and thus avoiding the enumeration of large numbers of compounds.^7,8^ Alternatively, several strategies that recently gained traction for boosting docking-based screening rely on iterative approaches utilizing machine learning (ML).^9−12^ The idea is simple, yet powerful: a small fraction of a large chemical library is docked by conventional means and used as training data for a ML model. The model then either classifies the remainder of the library in “virtual hits” and “non-hits” (DeepDocking^9^) or aims to predict the docking scores for the entire library (e.g., MolPAL,^10^ Glide Active Learning,^11^ and HASTEN^12^). This way, when not every compound needs to be docked, ML-boosted screening approaches can handle ultra-large libraries in a fraction of the time, providing the opportunity to explore the vast chemical landscape of giga-scale libraries.

ML acceleration with the help of regression models has, for instance, been previously achieved by a random forest (RF) model (option in MolPAL),^10^ an ensemble of RF and a Graph Convolutional Neural Network (Glide Active Learning),^11^ a simple feed-forward neural network (MolPAL),^10^ and by the message-passing neural network Chemprop (MolPAL^10^ and HASTEN^12^).

In this work, we rely on the tool HASTEN (macHine leArning booSTEd dockiNg), which uses Chemprop to predict docking scores in an iterative approach:^12^ in brief, HASTEN starts from an initial random compound selection from a large chemical library for a first conventional docking run and trains a ML model with the obtained data. Then, scores for the full compound library are predicted and used to rank all compounds. The best-ranked compounds next get selected for docking in the following iteration. This deliberate bias toward top-scoring compounds has been shown to result in excellent recall for data sets on the million scale.^12^ We aimed to investigate the applicability and performance of the HASTEN approach on even larger data sets, where the training data already reaches the million scale and predictions on the giga-scale cover billions of compounds.

To benchmark the performance of the ML-boosted approach on giga-scale chemical libraries, such libraries first need to be screened using standard brute-force docking to obtain the baseline data. Such docking data on the giga-scale has so far at least rarely been made publicly available: to date, the only example we are aware of are the Covid-19 screening results obtained with AutoDock-GPU on the Oak Ridge National Laboratory Summit computer.^13,14^

Herein, we used the fast Glide high-throughput virtual screening (HTVS) method in one of the largest conventional docking campaigns performed to date, to provide a brute-force docking baseline for analysis and comparison with our ML-boosted approach.^15^ We selected two distinct targets based on ongoing academic drug discovery projects.

The first target, the SurA protein, is a periplasmic chaperone found in Gram-negative bacteria. SurA has prolyl-peptidyl isomerase activity and is involved in the transport and maturation of several outer membrane proteins.^16−19^ Loss of SurA activity has been shown to render resistant bacterial strains sensitive to antibiotics, making SurA an interesting target in combating antibiotic resistance.^18,20^

Our second target was the cyclin G-associated kinase (GAK): a serine/threonine kinase, serving as a regulator of clathrin-mediated endocytosis and clathrin trafficking.^21−23^ GAK represents an important host factor involved in the regulation of viral entry and assembly of different RNA viruses, such as hepatitis C, dengue, and Ebola virus, and is of interest as an anti-viral target.^24−26^

Our study aims to benchmark and demonstrate the potential of ML-boosted screening with HASTEN for giga-scale applications, showcasing the speed-up, robustness, and successful recall of the majority of top-scoring compounds compared to the brute-force docking results for our two targets. We further demonstrate how large numbers of compounds that fail to dock successfully can hamper the HASTEN approach and discuss different options to handle such “failed” compounds, ultimately resulting in a novel screening protocol for HASTEN.

Finally, to support future screening approaches, we release a prepared version of the Enamine REAL lead-like screening library used in this study in a Glide-compatible format. Additionally, we release our full giga-scale docking results as benchmarking data sets for the future development and improvement of ML-boosted screening procedures.

## Approach

### Computational Infrastructure

Computational resources were provided by CSC—IT Center for Science Ltd.^27^ All calculations were performed on the CSC supercomputers Mahti (Atos BullSequana XH2000) and Puhti (Atos BullSequana X400), both running Red Hat Enterprise Linux Server release 7.9.

Ligand preparation and conventional docking steps were carried out with Mahti. Mahti features 1404 CPU nodes, each equipped with two AMD Rome 7H12 CPUs with 64 physical cores capable of two hardware threads running at 2.6 GHz base frequency and 256 GBs of system memory and Lustre parallel storage system providing a peak file I/O performance of 1.5 GB/s.

ML was conducted on CSC Puhti, which is equipped with 80 GPU nodes, in which each has four Nvidia Volta V100 GPUs. Puhti GPU nodes further feature two Intel Xeon Cascade Lake 20-core CPUs running at 2.1 GHz, 384 GB of system memory, and a local 3.6 TB NVMe disk.

### Screening Database Preparation

The Enamine REAL lead-like (ERLL) library, containing a total of 1.56 billion compounds (March 2021), was selected for the giga-scale screening.^5^ All included compounds have lead-like properties, with molecular weight ≤460 Da, Slog *P* −4.0 to 4.2, number of hydrogen bond acceptors <10 and donors <5, number of ring systems ≤4, and rotatable bonds ≤10. The library was obtained in the ChemAxon extended SMILES format and converted to regular SMILES using RDKit v2021.03.5, retaining the stereochemical information of the compounds where applicable.^28,29^

To reduce the variation in docking times between different subsets, compound order first was randomized, and SMILES were then evenly divided into 20 subsets. Ligand 3D structure preparation for docking was carried out with Schrödinger LigPrep (Schrödinger Suite 2021-1).^30^ Up to eight tautomers per compound and four stereoisomers per tautomer for a target pH of 7.0 ± 1.5 were generated, and compound geometries were energy-minimized with the OPLS_2005 forcefield.

Pre-prepared compounds were next collected into Schrödinger Phase databases for use during docking and future studies.^30−32^ Coordinates were stored in the compact internal coordinate representation, and a single conformation was generated with rapid sampling from each input compound during the phase revise step. To enable parallel processing within the CSC system wall time limits, the 20 input files were further split into a total of 781 individual phase databases of approx. 4.8 million compounds each.

### Receptor Preparation and SiteMap Analysis

Structures of SurA (PDB-ID 1M5Y, chain A)^33^ and GAK (PDB-ID 4Y8D, chain A)^34^ were retrieved from the RCSB Protein Data Bank.^35^ The Schrödinger Protein Preparation Wizard was used for structure preparation, hydrogen addition, and bond order assignment. Missing side chains were added with Prime.^30^ Crystallographic agents and water molecules were deleted. State generation for the original crystallographic ligand in the GAK structure was performed with Epik for pH 7.0 ± 2.0. Amino acid protonation states for pH 7.0 were assigned with PROPKA, and the hydrogen bonding network was optimized. Receptors were then subjected to a restrained energy minimization in the OPLS_2005 forcefield until the heavy atom rmsd compared to the previous minimization step fell below 0.3 Å.

For a comparison of the pocket properties of the two chosen targets, binding pocket properties were computed with Schrödinger SiteMap.^30,36,37^ The prepared GAK structure was directly subjected to SiteMap analysis, using only the site defined by the crystallographic ligand. SiteMap was run with default parameters (at least 15 site points per site, a more restrictive definition of hydrophobicity, standard grid, and cropping at 4 Å from the nearest site point).

The SurA *apo*-structure was first subjected to a 1 μs molecular dynamics simulation with Desmond and frames from the last 200 ns were analyzed with SiteMap to identify probable and druggable small molecule-binding sites (calculating up to five top-ranked sites per run; data not shown). The selected site was located in the crevice between N- and C-terminal core and P1 domains of SurA (for further discussion of the SurA domain architecture and pockets, see Calabrese et al.^38^).

### Receptor Grid Generation and Docking

Grid generation for SurA used the frame with the most druggable and consistently identified site, as described above (kindly provided by T. Kronenberger), and the receptor grid with a size of 30 Å^3^ was centered on the site centroid. For GAK, the grid center was defined as the centroid of the crystallographic ligand. Both grids were prepared with the OPLS_2005 forcefield. For GAK, additionally, a hydrogen-bonding constraint on the hinge-region amide (Cys126 backbone amide hydrogen) was set up.

Conventional docking of the 1.56 billion Enamine REAL lead-like library was carried out with Schrödinger Glide v9.0 in the HTVS mode.^15,30^ Van der Waals radii of nonpolar ligand atoms were scaled to 0.8 with a charge-cutoff of 0.15 e (default), and nonplanar amide conformations were penalized in both targets. Additionally, for GAK, the hydrogen-bonding constraint on the hinge-region amide of Cys126 was used. With the HTVS mode, the OPLS_2005 forcefield was used, and a single pose per ligand was collected after subjecting five poses to post-docking minimization.

### Simulated ML-Boosted Docking with HASTEN

The ML-boosted docking was simulated using the simu-dock mode in a local implementation of HASTEN v0.2 (optimized for CSC Puhti).^12^ For ML, Chemprop v1.3.1 (with Python 3.8.12) inside a singularity-container was used.^39^ Briefly, simu-dock allows the use of pre-generated docking data instead of actual docking in the HASTEN procedure. Whenever a compound is selected for docking by the algorithm, the pre-generated results of the conventional docking study will be loaded. Since scores are only added to the training data when compounds were selected for docking, the system exhibits identical behavior to screening with HASTEN when run including the brute-force docking steps.

In the first iteration, training was initialized with a random selection of 0.1% of the full library (1.56 million compounds). The selected compound subset was split randomly into training, validation, and test sets amounting to 80, 10, and 10% of the selected compounds, respectively. When no docking score was obtained during the conventional docking run (i.e., the compound did not dock successfully or failed to satisfy the constraint in GAK), an arbitrary failed score of +5.0 or 0.0 was applied, or failed compounds were excluded entirely from the training data. We performed one round of HASTEN with each treatment of failed compounds for each target. For SurA, experiments with a failed score of +5.0, and for GAK, experiments with the exclusion of failed compounds, were repeated in triplicate.

**Figure 1:**
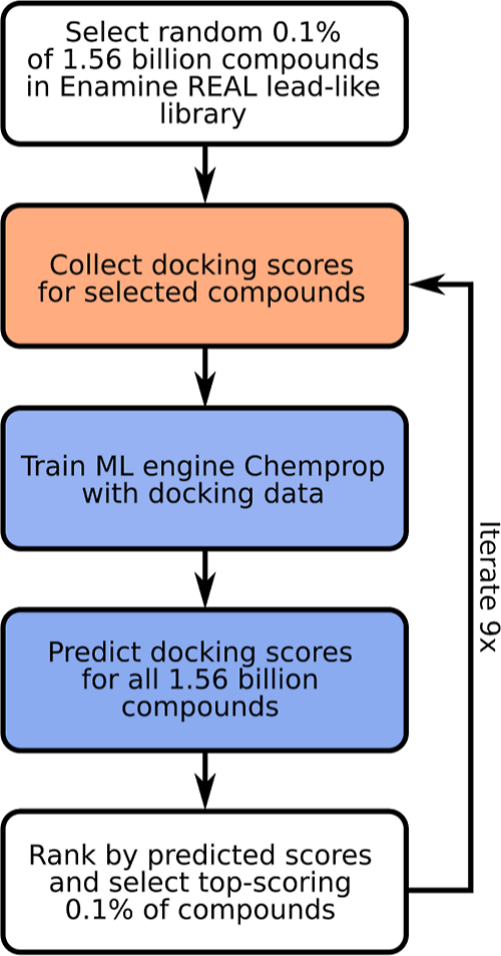
Overview of the HASTEN workflow for the ML-boosted giga-scale screening against SurA and GAK targets. In the conventional docking step (highlighted in orange), brute-force docking was simulated by loading the docking scores of selected compounds from the pre-generated brute-force docking data set. The ML procedure (highlighted in blue) involves a training step with all docking data of compounds that were selected for docking until the current iteration, followed by full giga-scale library prediction.

We used default parameters for regression in Chemprop to predict the docking score, except for the batch_size parameter, which was increased to 250 (from default: 50) to speed up training (see Supporting Information for a list of parameters). Once the training was completed, the scores for all 1.56 billion compounds in the library were predicted from their SMILES strings. Compounds were then ordered by the predicted score, and docking scores of the top-ranked 0.1% of compounds, that were not previously selected for docking, were loaded to simulate their conventional docking. All loaded docking scores were used to train the ML model from scratch during the next iteration, and the procedure was repeated nine times, which corresponds to docking 1% of the 1.56 billion input library by conventional means (compare also the schematic workflow in Figure 1).

Additionally, a HASTEN run with excluded failed compounds and a smaller docking fraction of only 0.01% per iteration was carried out for each target (all other settings were kept the same). This way, 10 iterations corresponded to docking only 0.1% of the 1.56 billion compounds. For GAK, the run with the reduced docking fraction was extended to a total of 25 iterations.

### Analysis of the Results

Recall values of the top 100, 1000, and 10,000 compounds were computed after every iteration. We define recall herein as the fraction of the true top-scoring 100, 1000, and 10,000 compounds when ranked by brute-force docking results, that were found using the ML model and selected for docking by HASTEN up until the current iteration.

Chemical similarities between the virtual hits and compounds in their training data set were evaluated using Tanimoto similarities calculated from 2048 bit Morgan fingerprints. Fingerprints were created using chemfp and RDKit, and Tanimoto similarities were calculated with chemfp.^29,40^

To assess the consistency of results obtained in repeated predictions, three replicates with a different random selection of compounds for the initial training set were used. The overlap of the compounds selected for docking by each model was calculated by counting which compounds among the top-scoring 100, 1000, and 10,000 compounds were selected by one, two, or three of the replicates after each iteration.

## Results and Discussion

### Chosen Targets SurA and GAK have Distinct Binding Pocket Properties

One objective in selecting targets for our case study was to ensure their distinct binding pocket properties. Since docking and, in particular, scoring success is target-dependent, any ML approach trained on docking scores will inherently reflect the same target dependence.^41^ In line with that, previous work with HASTEN, utilizing 12 literature targets, has already revealed different performances of the same protocol against different targets.^12^

However, having two targets with distinct properties assessed on the giga-scale would, for the first time, indicate whether the ML-boosted HASTEN performs equally well for both targets when benchmarked against the brute-force docking backdrop on the giga-scale and thus highlight whether potential additional dependencies arise from the choice of the ML method when employed on HASTEN’s intended use-scale. To assess the pocket properties, we first computed pocket descriptors with Schrödinger SiteMap, as summarized in Table 1.

**Table 1:** SurA and GAK Binding Pocket Properties Computed with SiteMap: DScore, Druggability Score (Values of 1.0 or Greater are Generally Considered Druggable);^37^ Hydrophobic, Hydrophilic, Hydrophobic, and Hydrophilic Character of the Site, Respectively (a Value of 1.0 Represents the Average for Tight-Binding Sites);^36^ don/acc, the Ratio of Hydrogen Bond Donors to Hydrogen Bond Acceptors

target	DScore	volume [Å^3^]	hydrophobic	hydrophilic	don/acc
SurA	1.15	363	1.52	0.80	1.25
GAK	1.03	507	0.66	1.02	0.91

The selected binding pocket of SurA was smaller than the GAK pocket (363 vs 507 Å^3^, Table 1) and displayed a high hydrophobicity (hydrophobic 1.52, Table 1). Furthermore, the binding site features more hydrogen bond donors than acceptors (donor acceptor ratio 1.25, Table 1). The GAK target was, on the other hand, more hydrophilic (hydrophilic 1.02, Table 1) and had a higher proportion of acceptors in the binding pocket (donor acceptor ratio 0.91, Table 1).

In conclusion, the two targets chosen for our case study display diverging binding pocket properties and can consequently be expected to favorably interact with different chemical scaffolds, thereby allowing us to investigate the method’s performance on the giga-scale in two distinct screening scenarios.

### Ready-to-Use Glide-Compatible Screening Library and Giga-Scale Brute-Force Docking to SurA and GAK

The first step toward the generation of a brute-force docking baseline was the preparation of ligand 3D structures from the original 1.56 billion input SMILES. Relevant tautomers and stereoisomers increased the library size to approx. 3.8 billion structures, which we distributed in 781 ligand databases for parallel docking. Preparation of this screening library took around 30 days (457,600 CPU hours) when utilizing 640 CPUs on the CSC Mahti supercomputer.

Next, we performed the brute-force docking of the complete prepared 3.8 billion structures to the SurA and GAK targets using Schrödinger Glide in the HTVS mode to generate the docking baseline for later comparison.^15^ With an approximate processing capacity of 40 compounds per minute per CPU core, Glide HTVS proved to be the fastest available method to generate a giga-scale docking data set. Glide HTVS gains its speed advantage from significantly cutting down conformational sampling compared to, for instance, the Glide Standard Precision (SP) protocol. This makes the approach more prone to false negatives, that is, compounds for which a good-scoring pose could have been found by more thorough sampling. On the other hand, compounds that score well with the HTVS protocol typically also score well with more extensive sampling, providing a meaningful selection of top-scoring hits. Importantly, before our screen, we confirmed the ability of the HTVS protocol to enrich true actives over decoys at least for the GAK target, where experimentally confirmed actives were already known (see section “GAK Receptor Selection and Method Validation” in the Supporting Information). Using 640 CPUs of the CSC Mahti supercomputer, we spent 85 days (1,305,216 CPU hours) on SurA docking, and the GAK conventional screening was completed in 53 days (809,216 CPU hours).

In our setting, the library preparation step contributed approx. 30% to the total required time for the full brute-force docking study. To support future screening efforts and enable a time reduction during the ligand preparation step, we release the entire prepared and randomized Enamine REAL lead-like library (March 2021) in 781 Glide-compatible, ready-to-use phase databases (https://doi.org/10.23729/2de314bb-59af-452a-955c-c2ff0c5ea57f). Moreover, we acknowledge that brute-force approaches to screening efforts on this scale remain elusive in most settings, even when using the fastest available docking methods. At the same time, giga-scale libraries are becoming more and more common and novel approaches to, for example, ML-boosted docking, should thus be evaluated on giga-scale data sets. In the hope of providing a useful benchmarking data set for such future applications, we further release our full giga-scale docking results for the two targets SurA and GAK.

(https://doi.org/10.23729/2170dc9c-4905-43c3-aeee-a574d360737f).

### ML-Boosted Giga-Scale Screening of SurA and GAK

For accelerating the screening process with the help of ML, we used the tool HASTEN, which has been previously validated with FRED and Glide SP docking results on the million scale.^12^

HASTEN aims to identify the top-scoring compounds rather than attempting a generalized prediction of docking scores for a given target. By iteratively selecting compounds with the best predicted scores, the training data will be progressively enriched in both true positives (already ranked correctly by the model) and false positives (ranked highly by the model, although the compounds dock poorly). This will improve HASTEN’s capability of identifying true top-scoring compounds with every iteration. Figure 1 summarizes our adapted procedure for the giga-scale screening.

We first started from an initial random selection of 0.1% of the 1.56 billion compounds. Previous work with HASTEN for million-scale data typically involved the selection of 1% of the library on each iteration, but since our training data at 0.1% already exceeds one million compounds, we decided to instead aim for a final total docking of 1% of the giga-scale library.

The brute-force docking step was simulated by utilizing the pre-generated docking data for the complete screening library: docking scores of compounds that were selected for docking by the algorithm were loaded directly. Only scoring data of selected compounds was considered, which ensures HASTEN to run as if the brute-force docking step had been performed as part of the workflow. Docking scores and corresponding compound SMILES were used as input data for training a ML model with Chemprop.^39^ Next, with the generated model, docking scores for the full ERLL library were predicted, and compounds were ranked by their predicted scores. During the next iteration, docking results for the top-ranked 0.1% of the compounds were added to the training data. This process was repeated nine times to end with a total training data set amounting to 1% of the full giga-scale data set.

Reducing the required number of compounds to dock to 1% of the full library lowered the total time spent on ligand preparation and SurA docking to around 1 day and 4 h when utilizing 640 CPUs of the CSC Mahti supercomputer and 20 h for the GAK target (including ligand preparation; 17,628 and 12,668 CPU hours for SurA and GAK, respectively).

The ML steps of the HASTEN protocol (Figure 1, blue boxes) consumed an additional 203–335 h for ML model training and prediction. Training took 143 h on a single Nvidia Volta V100 GPU of CSC Puhti. Prediction steps were distributed over 10 GPUs, resulting in a total prediction time of 52–184 h, depending on whether multiple predictions were run in parallel on one GPU or each GPU ran only a single instance of Chemprop. While a single instance per GPU was overall fastest, Chemprop, in our environment, utilized only a fraction of the available GPU compute power and memory, with its main bottleneck being compound featurization on the CPU. Thus, running four instances per GPU enabled higher throughput with the same hardware and constituted a better utilization of the computing resources on the supercomputer.

Removing the necessity of docking 99% of the entire giga-scale library allows the HASTEN procedure to complete the screening in around 10–14 days for each of the two targets. Additionally, depending on available resources, this approach enables a user to balance the computational load associated with a giga-scale screening project between CPUs (most docking tools) and the faster GPUs (for ML).

### Adjusting the Failed Score or Excluding Failed Compounds from the Training Data can Improve the Recall

When docking was unable to produce a docking score, for example because all sampled poses were energetically unfavorable, the original HASTEN protocol associated an arbitrary positive docking score with the affected SMILES strings to mimic a positive and therefore unfavorable energy. We started out with such a “failed score” of +5.0 for the SurA target and observed excellent recall values.

**Figure 2:**
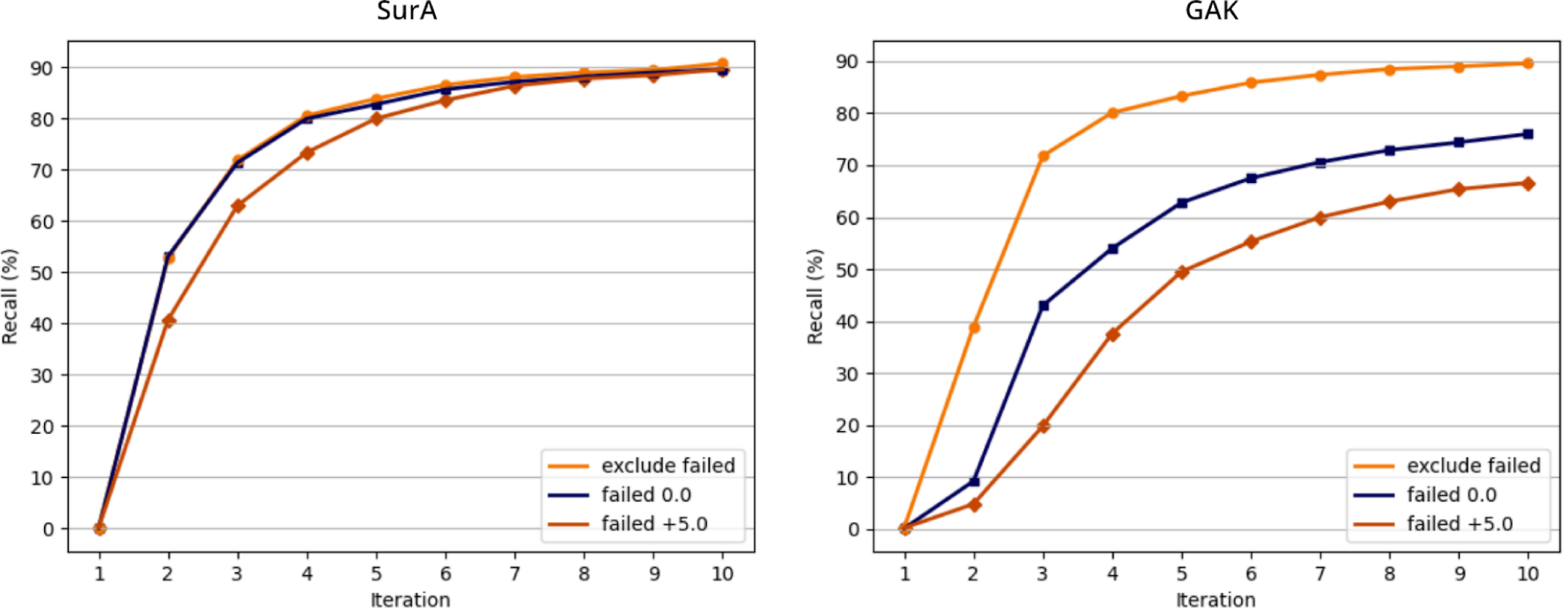
Recall curves of the 1000 top-scoring virtual hits in the HASTEN approach for SurA (left) and GAK (right) with different treatment of failed compounds: the curves represent the resulting recall per iteration for runs using a failed score of +5.0 (orange, diamonds), 0.0 (blue, squares), and excluding failed compounds from the training data (yellow, circles). The data are also summarized in Tables S1 and S2 in the Supporting Information.

Herein, we define recall as the number of true 100, 1000, and 10,000 top-scoring compounds according to the brute-force docking approach, that had also been selected for docking by HASTEN. With SurA and a failed score of +5.0, we were able to recall around 95, 90, and 85% of the top-scoring 100, 1000, and 10,000 compounds, respectively (see Figure 2 for top 1000 and Table S1). Using the same approach with the GAK target, on the other hand, resulted in recalls of only 70, 67, and 59% of the top-scoring 100, 1000, and 10,000 compounds, respectively (Figure 2 and Table S2).

One major difference between the data sets is the number of failed compounds: while for SurA less than 3% of all compounds in the ERLL library fails to dock (total failed: 42,149,150 compounds), for GAK, 45% are not docked successfully (704,564,272 compounds, compare also the overall distribution of docking scores in Figure S1). Consequently, a total of 46,846 failed compounds were selected into the HASTEN training data for SurA (final training data size >15 million compounds, of which 0.3% had the failed score). In contrast, for GAK, 1,471,958 failed compounds were part of the training data, that is around 9% of the total training data had the failed score of +5.0.

A reason for the high number of failing compounds may lie in the treatment of hydrogen-bonding constraints in Glide: with the active hinge region amide constraint, any compound with no hydrogen bond accepting group will be directly excluded from evaluation and receive the failed score. For any other compound, the initial placement will depend on its hydrogen bond acceptors: with constraint fulfillment being the first objective, rather than optimizing compound orientations for enclosure in the pocket, partially exposed compound poses can occur since they fulfill the constraint, albeit being energetically unfavorable and thus, likewise, receiving the failed score. The constraint thus forces compounds that would receive a favorable score when docked without the constraint to be evaluated in a different, more unfavorable receptor context.

We hypothesize that the large fraction of failed compounds in the training data drives the learning process in the ML step toward primarily identifying failed compounds to minimize the chosen metric (RMSE) rather than picking up smaller differences between the successfully docked compounds. Importantly, since failed compounds share features with those that dock successfully and because failure to dock can be an artifact of the docking methodology, the model can only imperfectly predict which compounds will fail. As a consequence, when their failed score is far outside the distribution of scores from successful dockings (see Figure S1), this will drive the model into associating higher, less favorable scores with features observed in failed compounds.

To improve the recall for the GAK data set, we next attempted to adjust the failed score parameter: motivated by our hypothesis, we set the failed score to 0.0 (closer to other obtained docking scores) to reduce the emphasis on failed compounds during training, which improved the recall of the top-scoring 100, 1000, and 10,000 compounds by 9–13% when compared to the failed score of +5.0 (compare Figure 2 and Table S2). We tested the same approach for SurA, which did, however, not consistently improve the recall (Table S1).

Finally, we assessed the complete removal of all failed compounds from the training data. Notably, the exclusion of all failed compounds resulted in a recall of 94, 90, and 84% of the top 100, 1000, and 10,000 true virtual hits for GAK, which is similar to the results achieved initially with SurA (compare Figure 2 and Tables S1 and S2). Moreover, using the same approach for SurA also improved the recall, albeit only slightly by about 1–2% (Table S1).

Comparing the number of failed compounds selected for docking during each iteration between the initial GAK screen with a failed score of +5.0 and the screen where failed compounds were excluded supports our hypothesis that the model learns to recognize compounds that will fail: the initial random selection of both runs includes around 708,000 failed compounds. In later iterations, only about 10% of this initial number get selected when failed compounds were considered with a failed score of +5.0 (see Figure S2). On the other hand, when excluding failed compounds completely and thus not providing the model with any training reference to recognize features of failed compounds, around 30–40% of the initial number of failed compounds get added during each iteration (see Figure S2).

When lowering the failed score or excluding failed compounds, the improvement of recall suggests that the learning process is instead driven by the identification of good-scoring compounds to minimize the RMSE metric. Furthermore, the validation and test set RMSE values per iteration suggest that a failed score closer to the mean or dropping of the failed compounds in both cases overall improves the model (see Figure S3).

In summary, we showed that in certain docking scenarios, the complete removal of failed compounds from the training data appears to improve the model quality. Depending on the case, it can also greatly enhance the recall (GAK) or show only a minor impact on the recalled compounds (SurA). In particular, dropping failed compounds is likely beneficial when a large proportion of evaluated compounds fail to dock successfully. This protocol modification can also add to the speed-up of model training as there is less data to process. Our case study identified the treatment of failed compounds or their assigned score as factors that can improve recall in ML-boosted screening campaigns with HASTEN/Chemprop.

### Prospects of Further Speed-up by Hyperparameter Choice and Protocol Modifications

A key motivation for our benchmarking effort and case study was to investigate the trade-off between maximizing speed and recall in a screening campaign with HASTEN. It is of note that all results presented herein were achieved with Chemprop default hyperparameters, and no hyperparameter optimization was performed. Given the size of our training data, any optimization would have to be done with similarly sized data and thus take a significant amount of additional time for every alternative explored.

While it is reasonable to assume that even better models could be achieved by careful optimization of the hyperparameters, the current work confirms that model quality and recall are good when utilizing the default Chemprop settings. Thus, default hyperparameters represent a suitable choice to harness the time-saving potential of ML-boosted docking with HASTEN.

In our test cases, we achieved highly similar RMSE values for validation and test sets (see Figure S3, Tables S3 and S4), suggesting good generalizability. It can further be seen that depending on the protocol and target, RMSE values of individual ML iterations converge well before the final iteration 10. Similarly, recall curves (see Figure 2) also often show an earlier convergence. Furthermore, the vast majority of the top compounds’ chemical diversity is already found on early iterations, and the predicted scores of the true top-scoring virtual hits are closely correlated even on earlier iterations, as shown exemplarily for the SurA target in Figures S4 and S5. Thus, if speed is of the essence, our results underline that HASTEN could, for example, be stopped already at iteration 5, which doubles the speed-up achieved by ML-boosted docking while often sacrificing less than 5% in recall of the top-scoring compounds.

This is further supported by a recent screening campaign performed by Orion Pharma, combining HASTEN with Glide SP to screen a 4.1 billion compound version of Enamine REAL against an oncology target. In this case, only two 0.1% iterations were performed and yielded approximately 100,000 high-scoring virtual hits (estimated recall based on the initial random sampling around 0.5, data not shown).^42^

Additional speed-up could likely be gained from the use of smaller docking fractions in the generation of the training data. To study this possibility, we re-ran HASTEN for both targets, this time adding only 0.01% of the compound library per iteration as training data (i.e., 156,000 compounds per iter.). Indeed, 10 iterations of these runs proved around 2–3 times faster than our original runs with a docking fraction of 0.1%. Recalls of 80, 68, and 62% of the true top 100, 1000, and 10,000 virtual hits were achieved for SurA, and 84, 73, and 60% for GAK, respectively (using the novel drop-failed protocol for both targets, see also Tables S5 and S6). While 10 iterations with a larger docking fraction allowed for higher recalls (SurA: 96, 91, and 87% and GAK: 94, 90, and 84% of the true top 100, 1000, and 10,000 virtual hits, respectively), the runs with smaller training data were still able to recall the bulk of the top-scoring compounds.

Arguably, a direct comparison by number of iterations is not the most suitable way to compare the two runs: for instance, when comparing recall relative to the number of total compounds docked, it becomes evident that iteratively docking the same number of compounds in smaller batches yields much higher recall than docking fewer, but larger, batches (see Figure S6 in the Supporting Information). This observation demonstrates the potential in utilizing smaller docking fractions with HASTEN. We herein assumed the same docking resources for both runs, but when resources for docking are limited, or when a slower docking method is chosen, opting for smaller docking fractions can represent a reasonable approach to keep the conventional docking at the necessary minimum.

However, another way to compare the models is to consider recall per runtime: when using a single Chemprop per GPU, the 0.1% docking fraction model can run for five iterations in slightly more than 7 days. This, in our setting, was faster than 10 iterations of the 0.01% docking fraction model (runtime approx. 8 days). Importantly, for SurA, the larger docking fraction recalls 92, 84, and 78% of the top-scoring 100, 1000, and 10,000 virtual hits in five iterations. Thus, the large model is faster, and its recall is 12–16% higher than with 10 iterations of the smaller docking fraction model (see Figure S7 and Table S5).

The difference is less dramatic when running multiple instances of Chemprop on each GPU during the prediction step: with parallelized Chemprop, for the large docking fraction-SurA model to consume less time than 10 iterations of the model with the small docking fraction, it can only run for three iterations (Table S5). In this case, its recalls of 81, 72, and 66% of the top-scoring 100, 1000, and 10,000 virtual hits are only slightly higher than with 10 iterations of the smaller docking fraction model (compare also Figure S8). For GAK, similarly, the model with the larger docking fraction would be faster when run for only 3–5 iterations with recalls being approximately on par or better by 5–15% (when stopped at iteration 3 or 5, respectively; see also Table S6, Figures S7 and S8). We continued our run with the smaller docking fraction for GAK for a total of 25 iterations to study whether recalls would eventually converge. With multiple Chemprop instances per GPU, this is still almost 2 days faster than 10 iterations of the large docking fraction model. Notably, on the final iteration, the recall of the top 100 virtual hits is lower by only 1%, and for the top 1000, by 5%, when only 3.9 million compounds were docked—less than during three iterations of the large fraction model.

The prediction step is a key reason why larger docking fractions can counter-intuitively be the faster choice: predictions consider all 1.56 billion compounds and thus take the same time irrespective of the docking fraction. Since every iteration features a prediction step, shortening the time spent on predictions from 18.4 h (single Chemprop/GPU) to 5.2 h (4 Chemprops/GPU) has been essential to harness the potential speed gain by the smaller docking fraction (compare Figures S7 and S8). Nonetheless, as the smaller model requires more iterations to reach the same recall and hence involves more prediction steps, choosing a larger docking fraction and fewer iterations represented a more optimal way to maximize the speed gain with the given resources in our hands.

In addition, our choice of a larger docking fraction was influenced by practical considerations: for instance, the size of our data exceeded 450 GB and was required to reside on fast NVMe storage (assigned to jobs at runtime) for performant operations in our environment. For every iteration, data had to be copied to the fast storage and moved back to permanent storage when the task was completed. Such copy operations become time-consuming in themselves at the data sizes faced with billions of compounds and it can thus be reasonable to limit the number of iterations also to reduce the necessary data shuffling.

We also noted that GPUs were more sought after in our computing environment and wait times in the queue were consequently often longer. Idle wait times between iterations can be another argument to opt for a larger docking fraction and fewer iterations. On the other hand, it is also possible to run the prediction step on CPU. For instance, a prediction run utilizing 1280 CPUs in our environment took 29.7 h. Thus, predictions on GPUs were around 2–6 times faster in our hands (depending on parallelization and number of utilized GPUs/CPUs). However, if CPUs are more easily accessible to a user in great bulk or utilizing CPUs cuts down idle wait times, prediction on CPUs is a suitable alternative. Taken together, depending on resource availability, HASTEN offers the user options to balance the computational load between CPUs (typically used for docking, also predictions at reduced speed) and GPUs (training and faster prediction) and we, herein, presented some examples of how to adapt the pipeline for a specific system and configuration.

We conclude that powerful predictive models can be obtained with Chemprop default hyperparameters and that a major time investment in parameter optimization is thus not strictly necessary. Our data highlight earlier stopping as a particularly promising approach to maximize screening speed-up, especially when utilizing large docking fractions. We also demonstrated the potential of smaller docking fractions, which, albeit in our hands overall providing lower recall (per runtime), recalled the majority of true virtual hits, and can thus help to adapt the HASTEN pipeline to scenarios where docking resources are limited. Finally, we outlined practical considerations, such as data shuffling and wall time limitations, and how they influenced our chosen approach. Thus, taking individual computational resources and practical implications into account, and depending on the emphasis of the screening campaign and the desired outcome, HASTEN runs can be tweaked to either maximize the speed or instead focus on maximally improving the recall.

### HASTEN Robustly Identifies the Same Compounds Irrespective of the Initial Random Selection

As a final step in our giga-scale assessment, we aimed to verify that HASTEN robustly recalls the majority of the top-scoring compounds irrespective of the initial random compound selection. To study the overlap of recalled compounds, we performed our ML-boosted screening experiment with a docking fraction of 0.1% per iteration in triplicate, with each run starting from a different random set of compounds.

**Figure 3:**
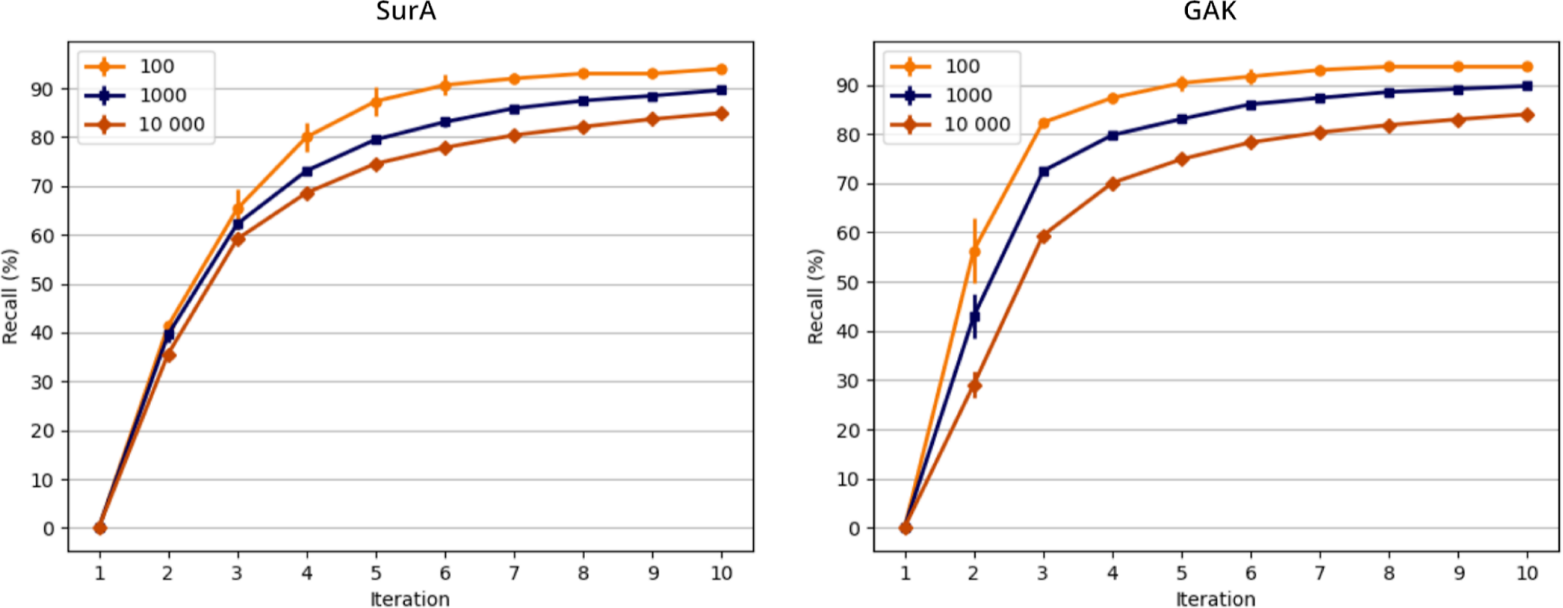
Recall curves for HASTEN runs performed in triplicate for the targets SurA (left, failed score +5.0) and GAK (right, failed compounds dropped). The recall is defined as the percentage of 100, 1000, and 10,000 top-scoring compounds according to the conventional docking, that were also selected for docking by the HASTEN approach. The percentage of top 100 virtual hits is shown in yellow (circles), top 1000 in blue (squares), and top 10,000 in orange (diamonds). The curves represent the average recall with error bars indicating the standard deviation. The individual results are listed in Tables S7 and S8 in the Supporting Information.

For SurA, we repeated the run with the original protocol and a failed score of +5.0, and for GAK, the run with failed compounds excluded from the training data. As can be seen in Figure 3, some variation occurs during the first iterations, especially for the top 100 and top 1000 virtual hits. However, recalls converged in the later iterations. The final recall of the top scoring 100, 1000, and 10,000 SurA virtual hits was on average 94, 90, and 85%, respectively. For GAK, average recalls were 94, 90, and 84% (see Tables S7 and S8). Thus, in conclusion, all three runs for both targets had a highly similar recall.

**Figure 4:**
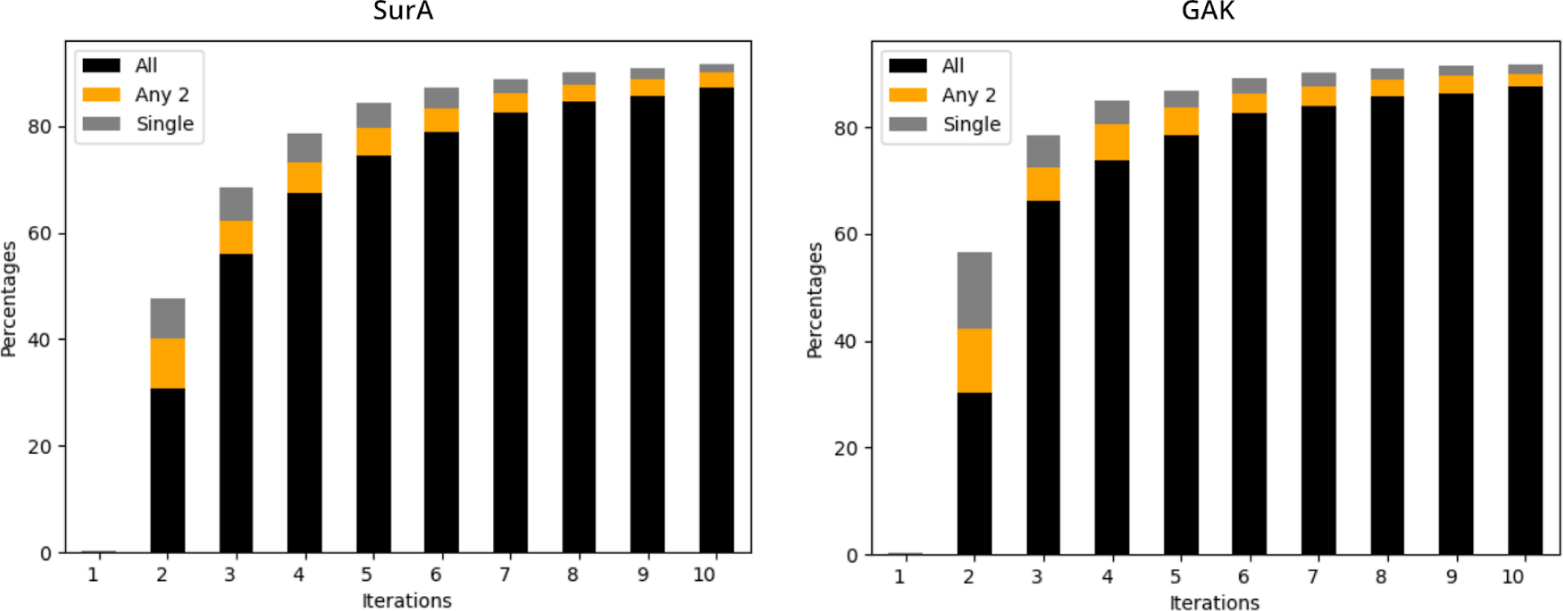
Overlap of top-scoring 1000 compounds recalled in three replicates of HASTEN runs for SurA (left) and GAK (right). Stacked bars indicate the percentage of recalled compounds per iteration, with black segments: compounds recalled in all three replicates; orange segments: compounds that were recalled by any two replicates; and gray segments: compounds that were only identified in a single run. The data are also visualized in Venn diagrams in Figures S9 and S10 in the Supporting Information for SurA and GAK, respectively.

We further investigated the overlap in compound selection: as shown in Figure 4 for the top 1000 virtual hits, the different HASTEN runs initially have no overlap, and from iteration 2 rapidly converge into largely the same final selection of compounds. Of the 90% recalled top 1000 virtual hits, around 30% were selected by all three replicates already on iteration 2 and around 87–88% were recalled consistently during the HASTEN runs by all three replicates (see Figure 4, black bars and Venn diagrams for iterations 2–10 in Figures S9 and S10 in the Supporting Information). Thus, our case study indicates that a single run of HASTEN is sufficient, and no major recall benefit could be gained from repeating experiments with a different initial random selection, at least in a setting with training data on the million scale. Additionally, the swift convergence into the same selection of compounds indicates that the robustness of the HASTEN approach can still be assumed when stopping on an earlier iteration.

## Conclusions

Our case study of the two targets SurA and GAK confirmed the applicability of the ML-boosted docking tool HASTEN on the so far unprecedented giga-scale. Our comparison with the corresponding brute-force docking results demonstrated comparable recall for the two distinct targets, identifying, for example, 90% of the true top-scoring 1000 virtual hits, although a modified protocol was necessary in the case of GAK. Given its benefit for the recall in both targets, this novel, modified protocol, which drops failed compounds from the training data, has now been made the default in HASTEN as of version 1.1.

Herein, our primary objective was the investigation and benchmarking of HASTEN for the speed-up of screening efforts on the giga-scale. While all tools that utilize ML for faster VS were ultimately developed to be able to process modern giga-scale screening libraries, to the best of our knowledge, so far no tool has been benchmarked against a billion-scale docking ground truth. Our work thus makes HASTEN the first application that underwent a benchmarking effort on the intended use-scale.

With the growth trend of chemical libraries in mind, novel approaches to facilitate giga-scale screening will likely continue to be developed. Benchmarking such approaches on ultra-large data sets remains challenging since the generation of giga-scale reference data is prohibited by time and resource consumption in many settings. We thus also release our full giga-scale docking results to provide others, seeking to predict docking scores from SMILES, with aptly sized benchmarking data sets.

Our “ground truth” and reference in this work were brute-force docking results generated with the particularly fast Glide HTVS protocol. Increased docking speed is typically achieved by limiting conformational sampling and is thus associated with a scoring accuracy trade-off and “false negatives”. The more robust a scoring protocol is in terms of fewer “mislabeled” compounds, the easier it should be for the ML model to associate a previously unseen compound structure with an appropriate score. In our hands, using the smaller 0.01% docking fraction of HTVS results did not achieve the same recall as with the larger docking fraction of 0.1%. However, as also backed by previous work with HASTEN and a small-scale comparison of Glide SP and HTVS results (data not shown), we conclude that equally powerful models to the ones achieved in this work could be generated with smaller training data stemming from more robust docking approaches, such as Glide SP. Importantly, this allows the approach to be tailored to the available resources: a user can either generate more training data with a less robust, but faster, docking methodology or rely on fewer docking results for training when utilizing more robust and computationally expensive methods.

One should keep in mind that ligand preparation and explicit brute-force docking steps remain the most time-consuming part of the procedure and should be kept to the required minimum. To support and potentially speed up future screening efforts involving brute-force docking campaigns, we release the entire prepared ERLL library as Glide-compatible, randomized ready-to-use screening databases.

Our results indicated additional time-saving potential in strategies such as earlier stopping, which we found often associated with only a minor drop in the recall of virtual hits. Depending on the available resources, as well as the desired outcome of the screening campaign, we herein outlined possible modifications to the HASTEN protocol for maximum speed-up or maximum recall of top-scoring hits.

It is important to note that we herein compare against brute-force docking as our baseline. Docking scores have known limitations in their ability to rank compounds correctly and identify true actives. When an approach can enrich actives over property-matched decoys on a small scale, this has been previously shown to translate to the ultra-large scale.^2^ For the GAK target, where experimentally confirmed actives were known, we validated the enrichment ability of the Glide HTVS protocol. It should be noted that the HASTEN protocol is, by definition, most useful when the chosen docking approach can, as in the case of GAK, successfully enrich in true actives.

In our case study, an excellent recall was achieved when using the default parameters of the ML engine Chemprop. It is however possible that both speed and recall could be further improved by performing hyperparameter optimization. While Chemprop provided excellent results in our case study, we acknowledge that it may not always be the ML engine of choice for every screening scenario.

In conclusion, HASTEN represents a robust approach to identify the bulk of the top-scoring virtual hits of a brute-force giga-scale docking campaign in a reduced time frame by reducing the required docking calculations by 99% (or more). In this work, HASTEN has been benchmarked on the giga-scale and shown to represent a viable strategy for ML-boosted docking to join a growing arsenal of methods designed to tackle the challenges associated with screening giga-scale libraries in everyday drug discovery.

## Data Availability

The full prepared ready-to-use Enamine REAL lead-like screening library (March 2021) in Schrödinger Phase database format is made available free of charge at https://doi.org/10.23729/2de314bb-59af-452a-955c-c2ff0c5ea57f. The final docking results for the two targets SurA and GAK are made available as giga-scale benchmarking data sets free of charge at https://doi.org/10.23729/2170dc9c-4905-43c3-aeee-a574d360737f. The tool HASTEN is freely available at https://github.com/TuomoKalliokoski/hasten.

## Supporting Information Available

Docking scores and selected failed compounds; validation and test set RMSEs; Tanimoto distances for SurA hits and training relatives; correlations of predicted scores on each iteration; summarized recalls; recall overlap for repeat calculations; summary of utilized Chemprop parameters; and extended methods: receptor selection and docking protocol validation for the GAK target (PDF)
Summary of lead-like GAK actives used for docking protocol validation as obtained from ChEMBL (XLSX)

## References

[ref1] Ferreira L. G.; Dos Santos R. N.; Oliva G.; Andricopulo A. D. Molecular Docking and Structure-Based Drug Design Strategies. Molecules 2015, 20, 13384–13421. 10.3390/molecules200713384. 26205061 PMC6332083

[ref2] Lyu J.; Wang S.; Balius T. E.; Singh I.; Levit A.; Moroz Y. S.; O’Meara M. J.; Che T.; Algaa E.; Tolmachova K.; Tolmachev A. A.; Shoichet B. K.; Roth B. L.; Irwin J. J. Ultra-large library docking for discovering new chemotypes. Nature 2019, 566, 224–229. 10.1038/s41586-019-0917-9. 30728502 PMC6383769

[ref3] Stein R. M.; Kang H. J.; McCorvy J. D.; Glatfelter G. C.; Jones A. J.; Che T.; Slocum S.; Huang X. P.; Savych O.; Moroz Y. S.; Stauch B.; Johansson L. C.; Cherezov V.; Kenakin T.; Irwin J. J.; Shoichet B. K.; Roth B. L.; Dubocovich M. L. Virtual discovery of melatonin receptor ligands to modulate circadian rhythms. Nature 2020, 579, 609–614. 10.1038/s41586-020-2027-0. 32040955 PMC7134359

[ref4] Warr W. A.; Nicklaus M. C.; Nicolaou C. A.; Rarey M. Exploration of Ultralarge Compound Collections for Drug Discovery. J. Chem. Inf. Model. 2022, 62, 2021–2034. 10.1021/acs.jcim.2c00224. 35421301

[ref5] Enamine REAL lead-like compounds. https://enamine.net/compound-collections/real-compounds/real-compound-libraries (accessed Aug 2, 2023).

[ref6] Kaplan A. L.; Confair D. N.; Kim K.; Barros-Álvarez X.; Rodriguiz R. M.; Yang Y.; Kweon O. S.; Che T.; McCorvy J. D.; Kamber D. N.; Phelan J. P.; Martins L. C.; Pogorelov V. M.; DiBerto J. F.; Slocum S. T.; Huang X. P.; Kumar J. M.; Robertson M. J.; Panova O.; Seven A. B.; Wetsel A. Q.; Wetsel W. C.; Irwin J. J.; Skiniotis G.; Shoichet B. K.; Roth B. L.; Ellman J. A. Bespoke library docking for 5-HT_2A_ receptor agonists with antidepressant activity. Nature 2022, 610, 582–591. 10.1038/s41586-022-05258-z. 36171289 PMC9996387

[ref7] Beroza P.; Crawford J. J.; Ganichkin O.; Gendelev L.; Harris S. F.; Klein R.; Miu A.; Steinbacher S.; Klingler F. M.; Lemmen C. Chemical space docking enables large-scale structure-based virtual screening to discover ROCK1 kinase inhibitors. Nat. Commun. 2022, 13, 6447 10.1038/s41467-022-33981-8. 36307407 PMC9616902

[ref8] Sadybekov A. A.; Sadybekov A. V.; Liu Y.; Iliopoulos-Tsoutsouvas C.; Huang X. P.; Pickett J.; Houser B.; Patel N.; Tran N. K.; Tong F.; Zvonok N.; Jain M. K.; Savych O.; Radchenko D. S.; Nikas S. P.; Petasis N. A.; Moroz Y. S.; Roth B. L.; Makriyannis A.; Katritch V. Synthon-based ligand discovery in virtual libraries of over 11 billion compounds. Nature 2022, 601, 452–459. 10.1038/s41586-021-04220-9. 34912117 PMC9763054

[ref9] Gentile F.; Agrawal V.; Hsing M.; Ton A. T.; Ban F.; Norinder U.; Gleave M. E.; Cherkasov A. Deep Docking: A Deep Learning Platform for Augmentation of Structure Based Drug Discovery. ACS Cent. Sci. 2020, 6, 939–949. 10.1021/acscentsci.0c00229. 32607441 PMC7318080

[ref10] Graff D. E.; Shakhnovich E. I.; Coley C. W. Accelerating high-throughput virtual screening through molecular pool-based active learning. Chem. Sci. 2021, 12, 7866–7881. 10.1039/d0sc06805e. 34168840 PMC8188596

[ref11] Yang Y.; Yao K.; Repasky M. P.; Leswing K.; Abel R.; Shoichet B. K.; Jerome S. V. Efficient Exploration of Chemical Space with Docking and Deep Learning. J. Chem. Theory Comput. 2021, 17, 7106–7119. 10.1021/acs.jctc.1c00810. 34592101

[ref12] Kalliokoski T. Machine Learning Boosted Docking (HASTEN): An Open-source Tool To Accelerate Structure-based Virtual Screening Campaigns. Mol. Inform. 2021, 40, 2100089 10.1002/minf.202100089. 34060239

[ref13] Acharya A.; Agarwal R.; Baker M. B.; Baudry J.; Bhowmik D.; Boehm S.; Byler K. G.; Chen S. Y.; Coates L.; Cooper C. J.; Demerdash O.; Daidone I.; Eblen J. D.; Ellingson S.; Forli S.; Glaser J.; Gumbart J. C.; Gunnels J.; Hernandez O.; Irle S.; Kneller D. W.; Kovalevsky A.; Larkin J.; Lawrence T. J.; LeGrand S.; Liu S. H.; Mitchell J. C.; Park G.; Parks J. M.; Pavlova A.; Petridis L.; Poole D.; Pouchard L.; Ramanathan A.; Rogers D. M.; Santos-Martins D.; Scheinberg A.; Sedova A.; Shen Y.; Smith J. C.; Smith M. D.; Soto C.; Tsaris A.; Thavappiragasam M.; Tillack A. F.; Vermaas J. V.; Vuong V. Q.; Yin J.; Yoo S.; Zahran M.; Zanetti-Polzi L. Supercomputer-Based Ensemble Docking Drug Discovery Pipeline with Application to Covid-19. J. Chem. Inf. Model. 2020, 60, 5832–5852. 10.1021/acs.jcim.0c01010. 33326239 PMC7754786

[ref14] Rogers D.; Glaser J.; Agarwal R.; Vermaas J.; Smith M.; Parks J.; Cooper C.; Sedova A.; Boehm S.; Baker M.; Smith J. SARS-CoV2 Docking Dataset, 2021. 10.1038/s41597-023-01984-9 PMC10044124 36977690

[ref15] Friesner R. A.; Banks J. L.; Murphy R. B.; Halgren T. A.; Klicic J. J.; Mainz D. T.; Repasky M. P.; Knoll E. H.; Shelley M.; Perry J. K.; Shaw D. E.; Francis P.; Shenkin P. S. Glide: a new approach for rapid, accurate docking and scoring. 1. Method and assessment of docking accuracy. J. Med. Chem. 2004, 47, 1739–1749. 10.1021/jm0306430. 15027865

[ref16] Tormo A.; Almirón M.; Kolter R. surA, an Escherichia coli gene essential for survival in stationary phase. J. Bacteriol. 1990, 172, 4339–4347. 10.1128/jb.172.8.4339-4347.1990. 2165476 PMC213259

[ref17] Rouvière P. E.; Gross C. A. SurA, a periplasmic protein with peptidyl-prolyl isomerase activity, participates in the assembly of outer membrane porins. Genes Dev. 1996, 10, 3170–3182. 10.1101/gad.10.24.3170. 8985185

[ref18] Lazar S. W.; Kolter R. SurA assists the folding of Escherichia coli outer membrane proteins. J. Bacteriol. 1996, 178, 1770–1773. 10.1128/jb.178.6.1770-1773.1996. 8626309 PMC177866

[ref19] Behrens S.; Maier R.; de Cock H.; Schmid F. X.; Gross C. A. The SurA periplasmic PPIase lacking its parvulin domains functions in vivo and has chaperone activity. EMBO J. 2001, 20, 285–294. 10.1093/emboj/20.1.285. 11226178 PMC140197

[ref20] Klein K.; Sonnabend M. S.; Frank L.; Leibiger K.; Franz-Wachtel M.; Macek B.; Trunk T.; Leo J. C.; Autenrieth I. B.; Schütz M.; Bohn E. Deprivation of the Periplasmic Chaperone SurA Reduces Virulence and Restores Antibiotic Susceptibility of Multidrug-Resistant Pseudomonas aeruginosa. Front. Microbiol. 2019, 10, 100 10.3389/fmicb.2019.00100. 30846971 PMC6394205

[ref21] Kanaoka Y.; Kimura S. H.; Okazaki I.; Ikeda M.; Nojima H. GAK: a cyclin G associated kinase contains a tensin/auxilin-like domain. FEBS Lett. 1997, 402, 73–80. 10.1016/s0014-5793(96)01484-6. 9013862

[ref22] Zhang C. X.; Engqvist-Goldstein A. E. Y.; Carreno S.; Owen D. J.; Smythe E.; Drubin D. G. Multiple Roles for Cyclin G-Associated Kinase in Clathrin-Mediated Sorting Events. Traffic 2005, 6, 1103–1113. 10.1111/j.1600-0854.2005.00346.x. 16262722

[ref23] Sorrell F. J.; Szklarz M.; Abdul Azeez K.; Elkins J. M.; Knapp S. Family-wide Structural Analysis of Human Numb-Associated Protein Kinases. Structure 2016, 24, 401–411. 10.1016/j.str.2015.12.015. 26853940 PMC4780864

[ref24] Neveu G.; Barouch-Bentov R.; Ziv-Av A.; Gerber D.; Jacob Y.; Einav S. Identification and Targeting of an Interaction between a Tyrosine Motif within Hepatitis C Virus Core Protein and AP2M1 Essential for Viral Assembly. PLoS Pathog. 2012, 8, e1002845 10.1371/journal.ppat.1002845. 22916011 PMC3420927

[ref25] Neveu G.; Ziv-Av A.; Barouch-Bentov R.; Berkerman E.; Mulholland J.; Einav S. AP-2-Associated Protein Kinase 1 and Cyclin G-Associated Kinase Regulate Hepatitis C Virus Entry and Are Potential Drug Targets. J. Virol. 2015, 89, 4387–4404. 10.1128/jvi.02705-14. 25653444 PMC4442395

[ref26] Bekerman E.; Neveu G.; Shulla A.; Brannan J.; Pu S.-Y.; Wang S.; Xiao F.; Barouch-Bentov R.; Bakken R. R.; Mateo R.; Govero J.; Nagamine C. M.; Diamond M. S.; De Jonghe S.; Herdewijn P.; Dye J. M.; Randall G.; Einav S. Anticancer kinase inhibitors impair intracellular viral trafficking and exert broad-spectrum antiviral effects. J. Clin. Invest. 2017, 127, 1338–1352. 10.1172/jci89857. 28240606 PMC5373883

[ref27] CSC–IT Center for Science Ltd. https://www.csc.fi (accessed Feb 06, 2023).

[ref28] ChemAxon Extended SMILES and SMARTS - CXSMILES and CXSMARTS. https://docs.chemaxon.com/display/docs/chemaxon-extended-smiles-and-smarts-cxsmiles-and-cxsmarts.md (accessed August 24, 2023).

[ref29] RDKit: Open-source cheminformatics. http://www.rdkit.org (accessed March 30, 2021).

[ref30] Schrödinger, LLC. Schrödinger Suite 2021-1. Epik v5.5. Glide v9.0, LigPrep. Phase. Prime. Protein Preparation Wizard. SiteMap: New York, NY, 2021.

[ref31] Dixon S. L.; Smondyrev A. M.; Knoll E. H.; Rao S. N.; Shaw D. E.; Friesner R. A. PHASE: a new engine for pharmacophore perception, 3D QSAR model development, and 3D database screening: 1. Methodology and preliminary results. J. Comput. Aided Mol. Des. 2006, 20, 647–671. 10.1007/s10822-006-9087-6. 17124629

[ref32] Dixon S. L.; Smondyrev A. M.; Rao S. N. PHASE: A Novel Approach to Pharmacophore Modeling and 3D Database Searching. Chem. Biol. Drug Des. 2006, 67, 370–372. 10.1111/j.1747-0285.2006.00384.x. 16784462

[ref33] Bitto E.; McKay D. B. Crystallographic structure of SurA, a molecular chaperone that facilitates folding of outer membrane porins. Structure 2002, 10, 1489–1498. 10.1016/s0969-2126(02)00877-8. 12429090

[ref34] Kovackova S.; Chang L.; Bekerman E.; Neveu G.; Barouch-Bentov R.; Chaikuad A.; Heroven C.; Šála M.; De Jonghe S.; Knapp S.; Einav S.; Herdewijn P. Selective Inhibitors of Cyclin G Associated Kinase (GAK) as Anti-Hepatitis C Agents. J. Med. Chem. 2015, 58, 3393–3410. 10.1021/jm501759m. 25822739 PMC4431592

[ref35] Berman H. M.; Westbrook J.; Feng Z.; Gilliland G.; Bhat T. N.; Weissig H.; Shindyalov I. N.; Bourne P. E. The Protein Data Bank. Nucleic Acids Res. 2000, 28, 235–242. 10.1093/nar/28.1.235. 10592235 PMC102472

[ref36] Halgren T. New Method for Fast and Accurate Binding-site Identification and Analysis. Chem. Biol. Drug Des. 2007, 69, 146–148. 10.1111/j.1747-0285.2007.00483.x. 17381729

[ref37] Halgren T. A. Identifying and characterizing binding sites and assessing druggability. J. Chem. Inf. Model. 2009, 49, 377–389. 10.1021/ci800324m. 19434839

[ref38] Calabrese A. N.; Schiffrin B.; Watson M.; Karamanos T. K.; Walko M.; Humes J. R.; Horne J. E.; White P.; Wilson A. J.; Kalli A. C.; Tuma R.; Ashcroft A. E.; Brockwell D. J.; Radford S. E. Inter-domain dynamics in the chaperone SurA and multi-site binding to its outer membrane protein clients. Nat. Commun. 2020, 11, 2155 10.1038/s41467-020-15702-1. 32358557 PMC7195389

[ref39] Yang K.; Swanson K.; Jin W.; Coley C.; Eiden P.; Gao H.; Guzman-Perez A.; Hopper T.; Kelley B.; Mathea M.; Palmer A.; Settels V.; Jaakkola T.; Jensen K.; Barzilay R. Analyzing Learned Molecular Representations for Property Prediction. J. Chem. Inf. Model. 2019, 59, 3370–3388. 10.1021/acs.jcim.9b00237. 31361484 PMC6727618

[ref40] Dalke A. The chemfp project. J. Cheminf. 2019, 11, 76 10.1186/s13321-019-0398-8. PMC6896769 33430977

[ref41] Yuriev E.; Holien J.; Ramsland P. A. Improvements, trends, and new ideas in molecular docking: 2012-2013 in review. J. Mol. Recognit. 2015, 28, 581–604. 10.1002/jmr.2471. 25808539

[ref42] Kalliokoski T.; Turku A. HASTENing structure-based virtual screening of large chemical libraries. The 12th International Conference on Chemical Structures: Noordwijkerhout, The Netherlands, 2022. https://drive.google.com/file/d/1wKJzOsjut_4KEv5MNhe0YP9FRyAuBLi_/view .

