# Supplementary material for: Machine Learning-Boosted Docking Enables the Efficient Structure-Based Virtual Screening of Giga-Scale Enumerated Chemical Libraries
Source: J Chem Inf Model. 2023 Sep 1;63(18):5773–83. doi: 10.1021/acs.jcim.3c01239 (PMC10523430; doi:10.1021/acs.jcim.3c01239)
Supplement: Supplementary file 1 — ci3c01239_si_001.pdf [file ci3c01239_si_001.pdf]

Supporting Information for:

Machine Learning-Boosted Docking Enables the Efficient Structure-Based Virtual Screening of Giga-Scale Enumerated Chemical Libraries

Toni Sivula,[†] Laxman Yetukuri,[‡] Tuomo Kalliokoski,[¶] Heikki Käsnänen,[¶] Antti Poso,[†,§] and Ina Pöhner[*,†]

†*School of Pharmacy, University of Eastern Finland, FI-70211 Kuopio, Finland*

‡*CSC – IT Center for Science Ltd., FI-02101 Espoo, Finland*

¶*Computational Medicine Design, Orion Pharma, Orionintie 1A, FI-02101 Espoo, Finland*

§*Department of Internal Medicine VIII, University Hospital Tübingen, DE-72076 Tübingen, Germany*

E-mail: ina.pohner@uef.fi

Contents

Supplementary figures 3

Figure S1: Distribution of docking scores for SurA and GAK datasets 3

Figure S2: Failed compounds selected for docking per HASTEN iteration for GAK target 4

Figure S3: Validation and test set RMSE curves for SurA and GAK 5

Figure S4: Tanimoto distance boxplots for SurA hits and closest training relatives 6

Figure S5: Correlations of predicted scores per iteration for virtual hits of SurA 7

Figure S6: Recalls for docking fractions 0.1% and 0.01% vs. docked compounds 8

Figure S7: Recalls for docking fractions 0.1% and 0.01% vs. time (single Chemprop) . . 9

Figure S8: Recalls for docking fractions 0.1% and 0.01% vs. time (multiple Chemprops) 10

Figure S9: Venn diagrams showing recall overlap of top 1000 virtual hits for SurA target 11

Figure S10: Venn diagrams showing recall overlap of top 1000 virtual hits for GAK target 12

Supplementary tables 13

Table S1: Recalls for SurA with failed compounds scored as +5, 0 or dropped 13

Table S2: Recalls for GAK with failed compounds scored as +5, 0 or dropped 14

Table S3: Validation and test set RMSE per Chemprop training iteration for SurA 15

Table S4: Validation and test set RMSE per Chemprop training iteration for GAK 16

Table S5: Recalls and runtime of runs with 0.1% and 0.01% docking fraction for SurA . 17

Table S6: Recalls and runtime of runs with 0.1% and 0.01% docking fraction for GAK . 18

Table S7: Recalls of top 100, 1000, and 10 000 virtual hits for SurA target 19

Table S8: Recalls of top 100, 1000, and 10 000 virtual hits for GAK target 20

Selected Chemprop parameters 21

GAK receptor selection and method validation 22

Supplementary figures

Figure S1: Bar plots illustrating the distribution of docking scores for the entire 1.56 billion ERLL library brute-force docking results in the targets SurA (left) and GAK (right). Docking scores were rounded to their nearest integer value and converted to percentages relative to the size of the complete ERLL library. Any compound for which no docking score was obtained is represented with the HASTEN protocol failed score of +5.0.

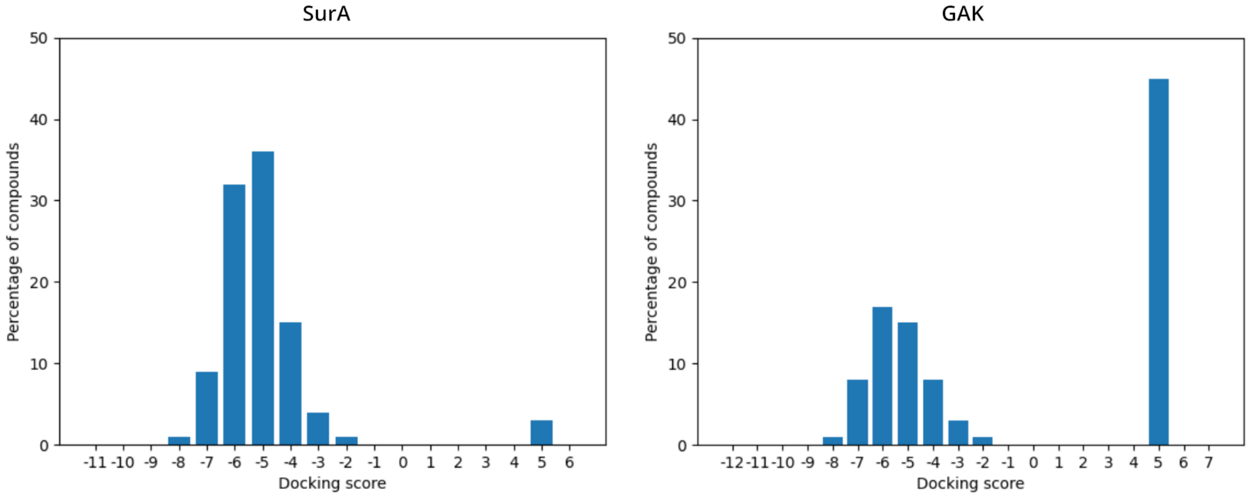

Figure S2: Bar plots showing the number of failed compounds selected for docking on each HASTEN iteration for the GAK target when using a failed score of +5.0 (blue) and excluding failed compounds from the training data (orange).

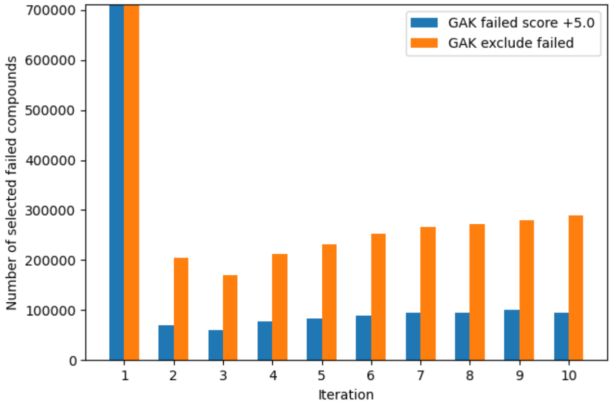

Figure S3: Validation and test set RMSE per Chemprop training iteration for the targets SurA (left) and GAK (right) with different treatments of failed compounds: Validation set RMSE curves are shown semi-translucent and, if invisible, are overlaid by the test set RMSE curves due to highly similar RMSEs. The data is also summarized in Tables **S3** and **S4**. RMSEs are shown for HASTEN runs utilizing a failed score of +5.0 (orange, diamonds) or 0.0 (blue, squares), and for runs that excluded failed compounds from the training data (yellow, circles). For SurA, the average validation and test set RMSE values per iteration are shown for the three replicates using a failed score of +5.0, and for GAK, the average of three replicates where failed compounds were dropped.

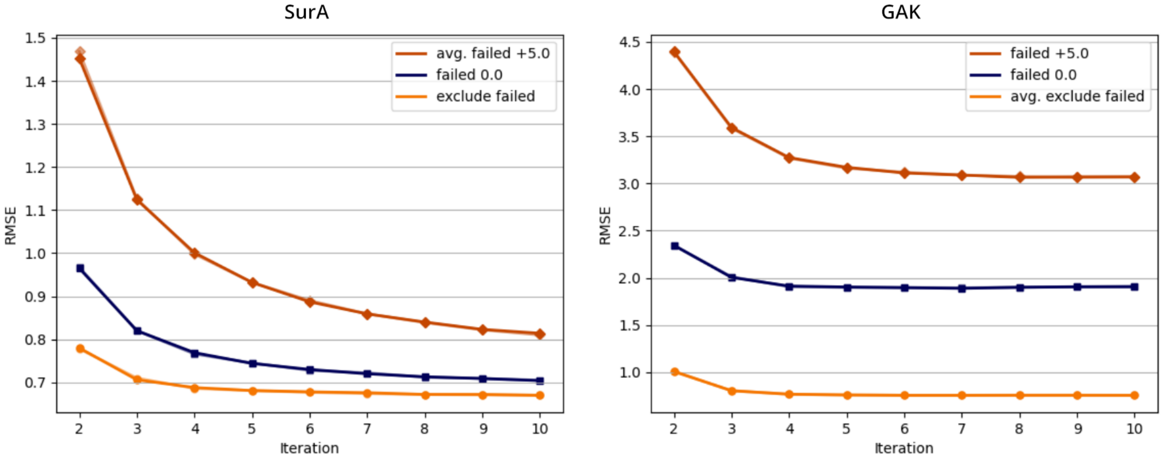

Figure S4: Boxplots of Tanimoto distances of top-scoring virtual hits for SurA to their closest training dataset relative (i.e. the largest Tanimoto similarity to any compound that was selected on a previous iteration). The data shown is for all compounds among the true top 10 000 virtual hits that were selected by HASTEN and was obtained with the help of chemfp from the run with a docking fraction of 0.1% per iteration and the drop-failed protocol.

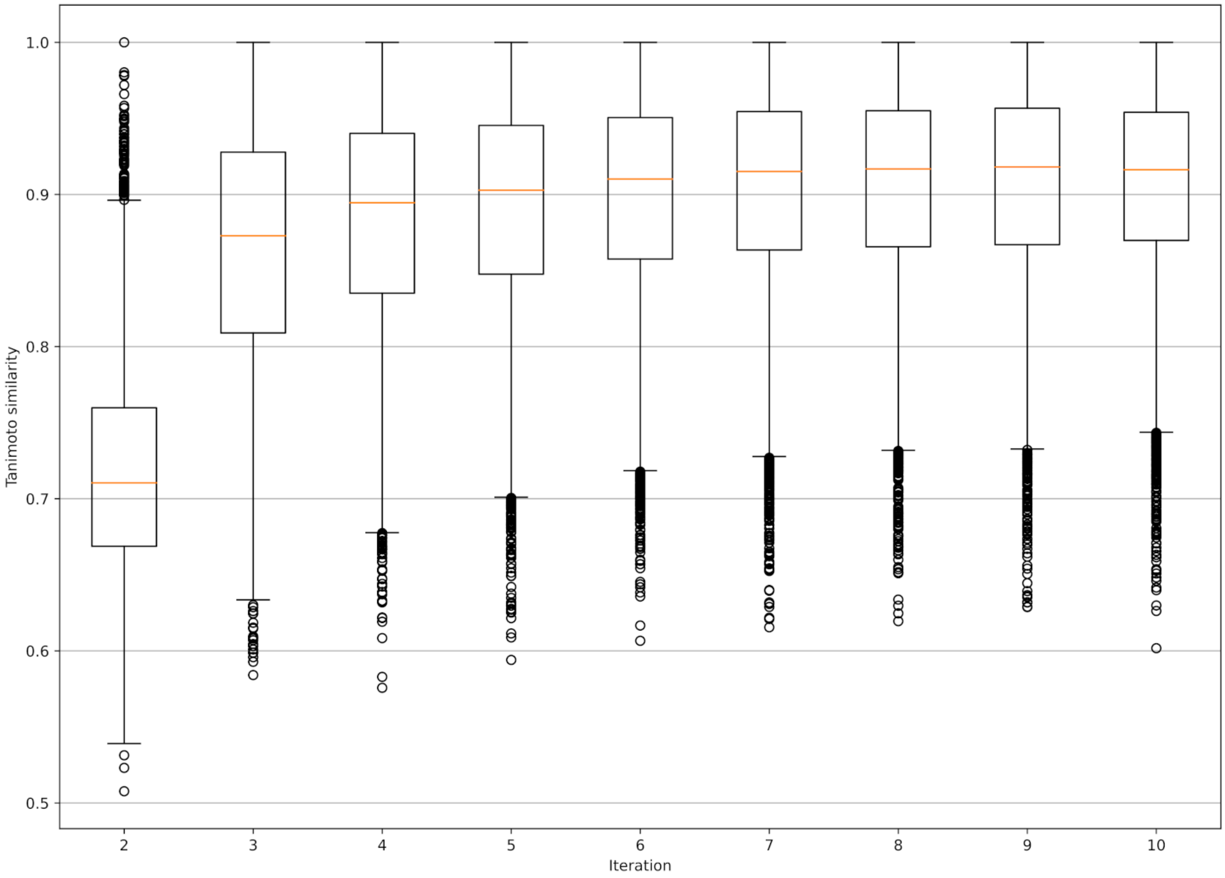

Figure S5: Heatmap of Pearson correlations of predicted scores on iterations 2-10 for the SurA top virtual hits (defined here by a docking score cutoff of -9.0, total: 37 818 compounds). The analyzed models were obtained with a docking fraction of 0.1% per iteration and the drop-failed protocol.

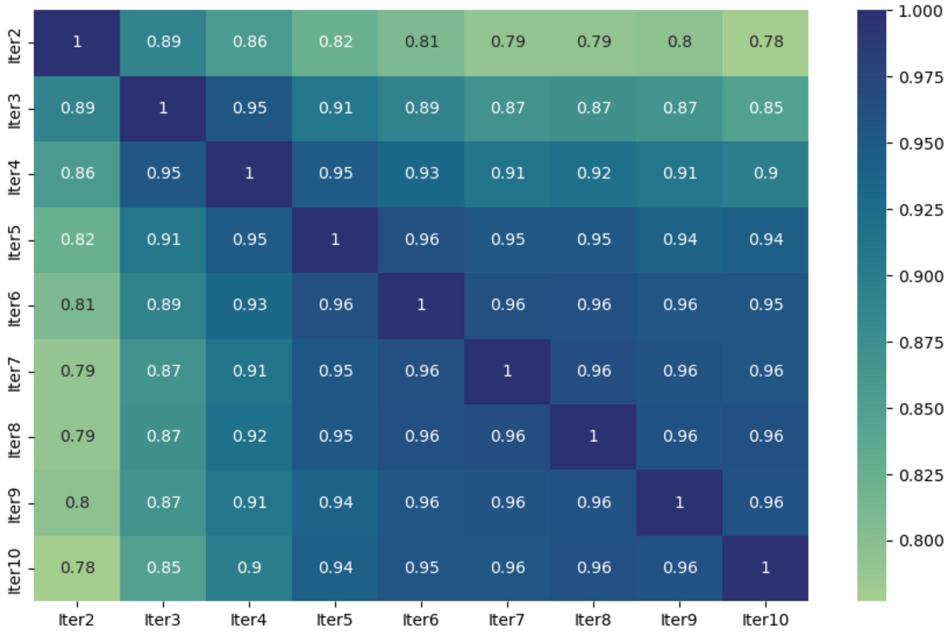

Figure S6: Recalls of the top 100 (top), 1000 (middle) and 10 000 (bottom) true virtual hits in the runs with docking fractions of 0.1% (orange) and 0.01% (blue) expressed as a function of the total number of compounds docked. The left column shows results for the SurA target and the right column, GAK. All HASTEN runs shown in this plot were done with the drop-failed protocol.

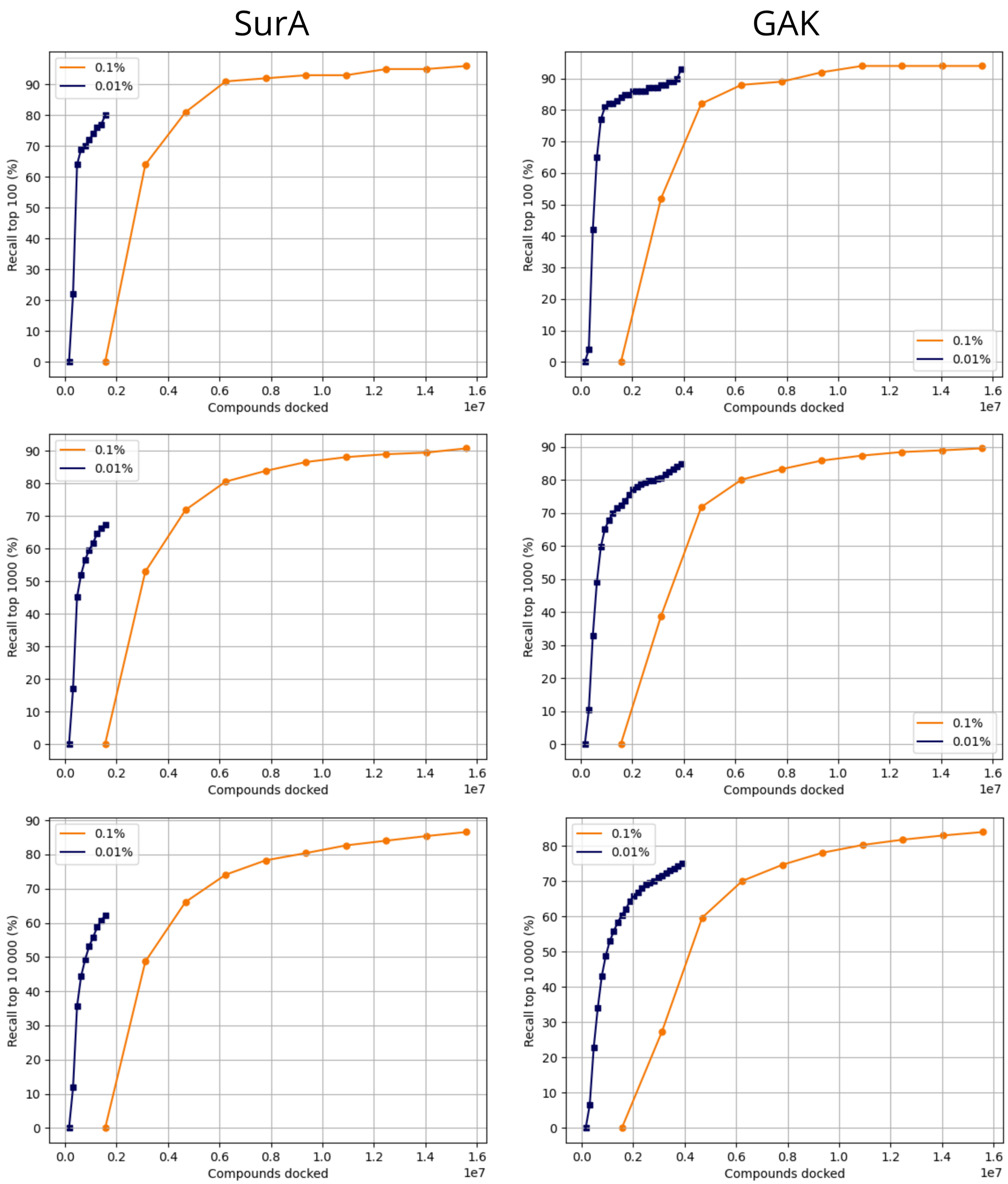

Figure S7: Recalls of the top 100 (top), 1000 (middle) and 10 000 (bottom) true virtual hits in the runs with docking fractions of 0.1% (orange) and 0.01% (blue) expressed as a function of the total runtime in minutes when predictions ran with a single Chemprop per GPU. The left column shows results for the SurA target and the right column, GAK. All HASTEN runs shown in this plot were done with the drop-failed protocol.

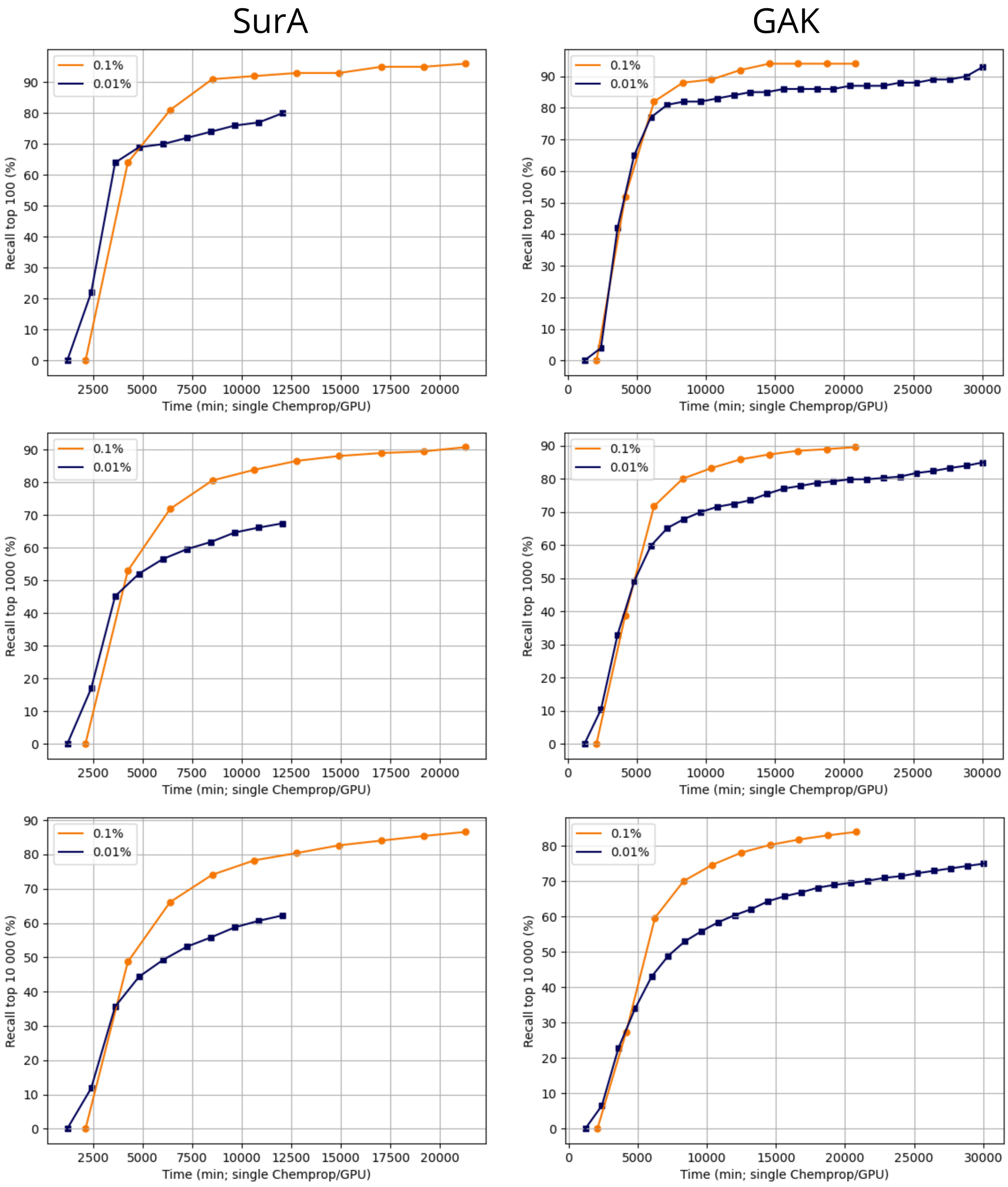

Figure S8: Recalls of the top 100 (top), 1000 (middle) and 10 000 (bottom) true virtual hits in the runs with docking fractions of 0.1% (orange) and 0.01% (blue) expressed as a function of the total runtime in minutes when predictions ran with 4 Chemprops per GPU. The left column shows results for the SurA target and the right column, GAK. All HASTEN runs shown in this plot were done with the drop-failed protocol.

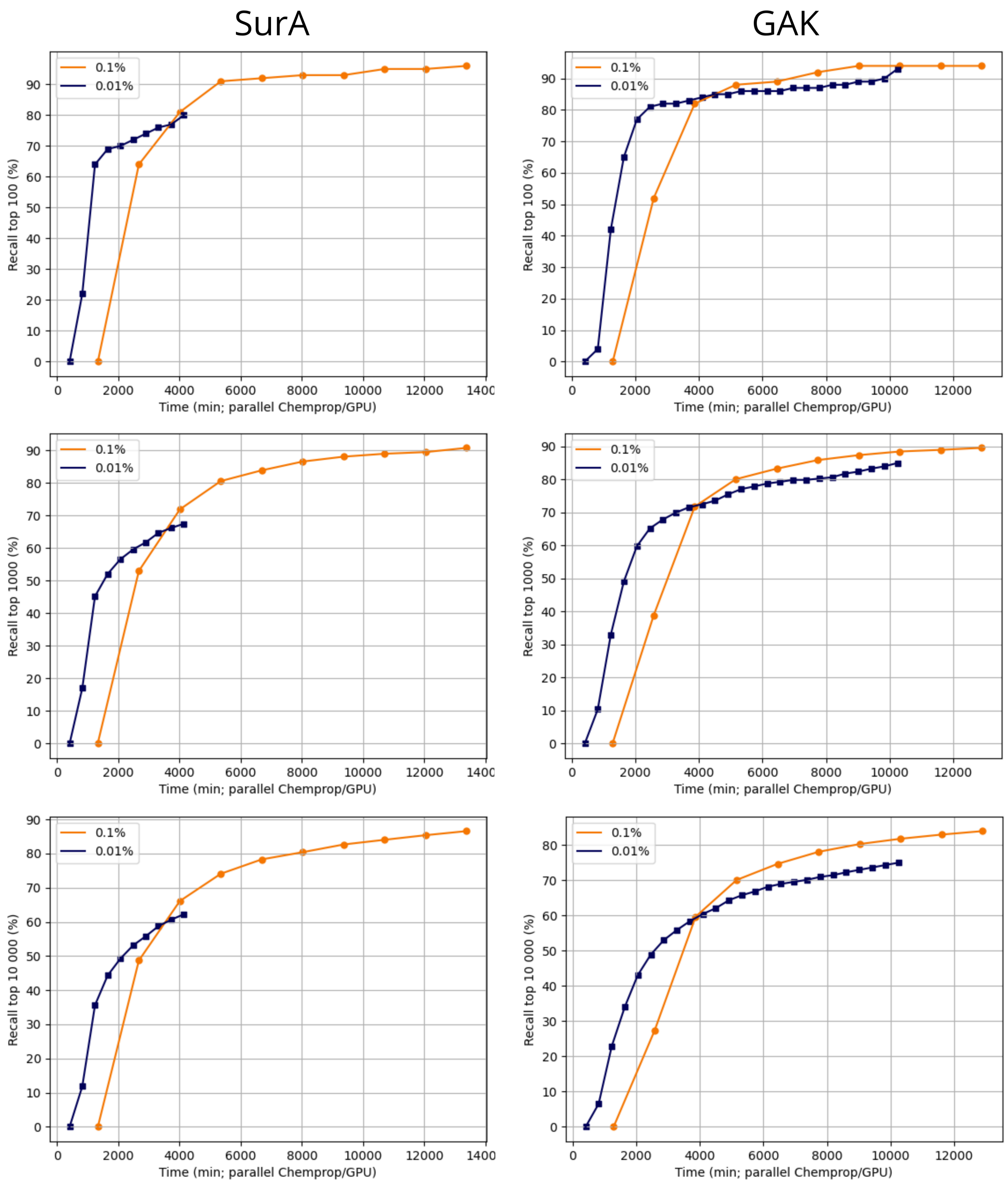

Figure S9: Venn diagrams illustrating the overlap in recalled compounds among the top 1000 virtual hits for the SurA target. Compound numbers are shown for each replicate R on every HASTEN iteration with a failed score of +5.0, starting from iteration 2.

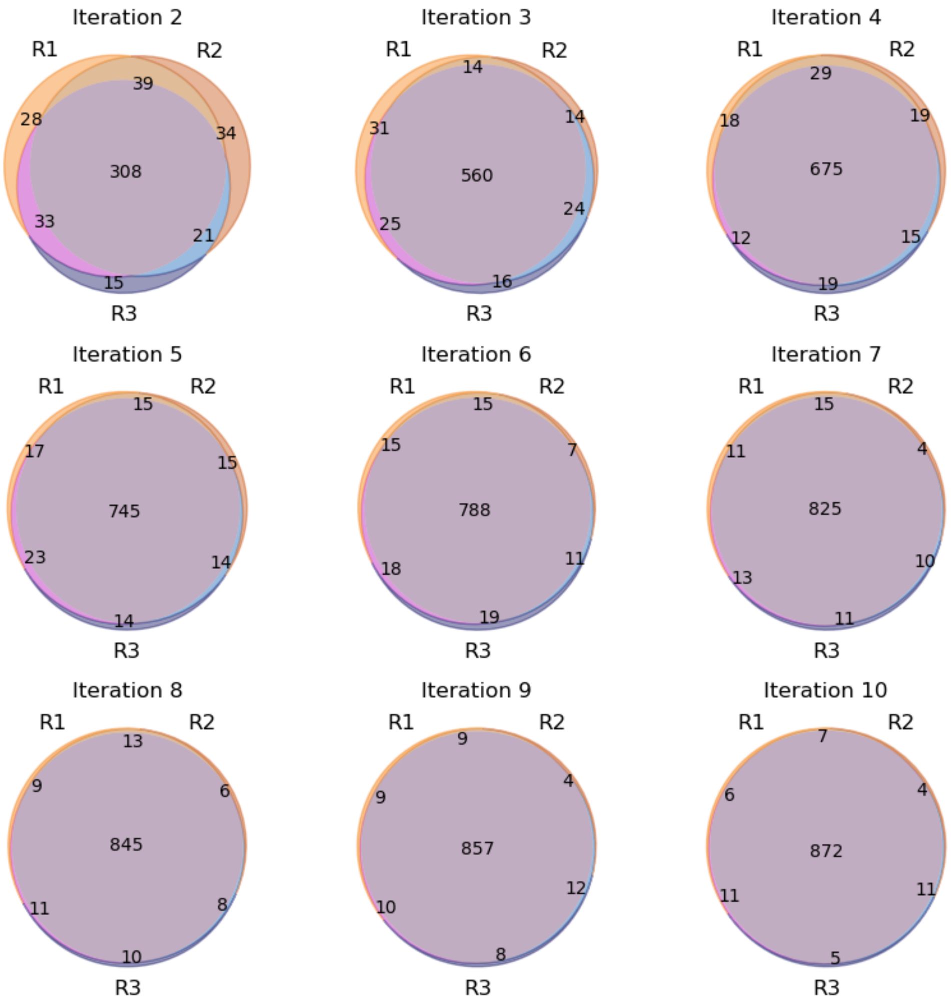

Figure S10: Venn diagrams illustrating the overlap in recalled compounds among the top 1000 virtual hits for the GAK target. Compound numbers are shown for each replicate R on every HASTEN iteration with excluded failed compounds, starting from iteration 2.

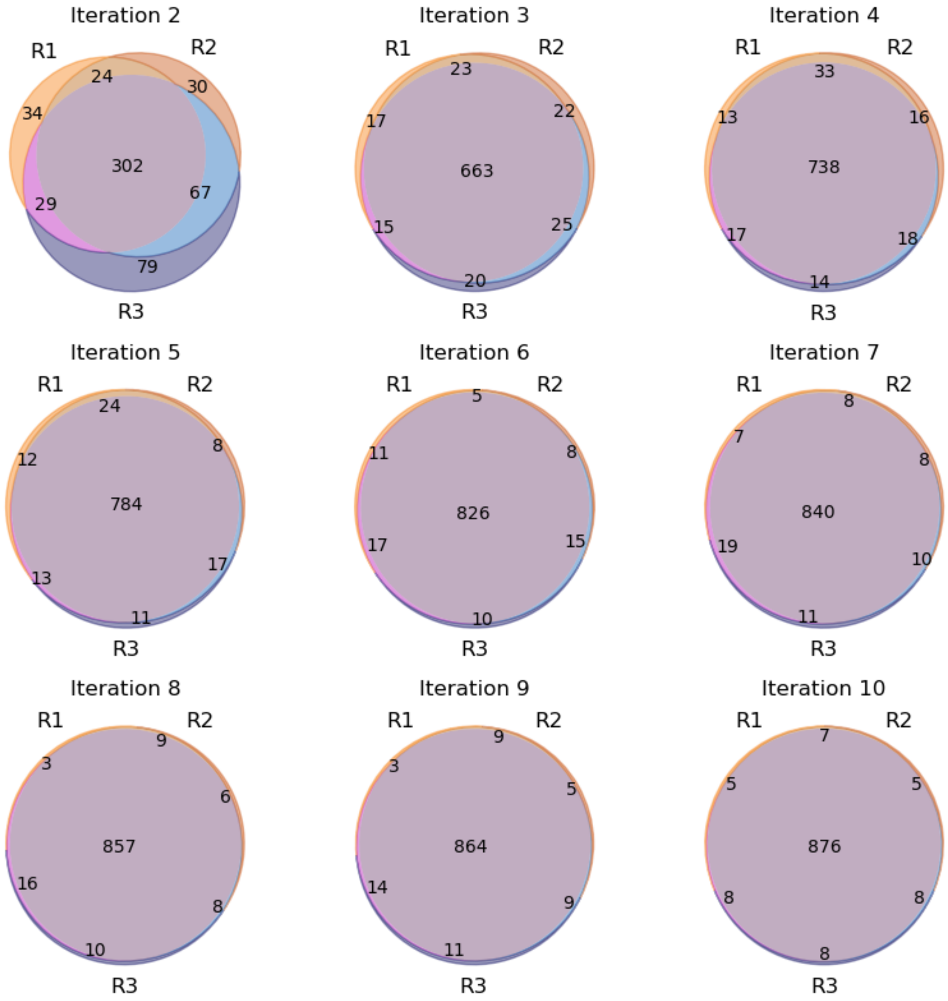

Supplementary tables

Table S1: Recalls for the target SurA with failed compounds scored as +5.0, 0.0 or dropped. For the failed score of +5.0, only the first replicate run is reported.

	Top 100			Top 1000			Top 10000		
Iteration	+5.0	0.0	exclude	+5.0	0.0	exclude	+5.0	0.0	exclude
1	0	0	0	1	0	2	13	8	10
2	42	58	64	408	531	530	3675	4784	4868
3	70	80	81	630	714	719	5970	6536	6609
4	83	88	91	734	800	806	6877	7334	7408
5	90	90	92	800	828	839	7486	7711	7829
6	93	92	93	836	857	866	7806	8017	8040
7	93	92	93	864	872	881	8045	8213	8265
8	94	93	95	878	882	890	8223	8365	8403
9	94	93	95	885	890	895	8340	8510	8537
10	95	93	96	896	896	908	8477	8627	8657

Table S2: Recalls for the target GAK with failed compounds scored as +5.0, 0.0 or dropped. For the run where failed compounds were dropped, only the first replicate run is reported.

	Top 100			Top 1000			Top 10000		
Iteration	+5.0	0.0	exclude	+5.0	0.0	exclude	+5.0	0.0	exclude
1	0	0	0	2	1	2	13	12	17
2	1	7	52	48	92	389	450	828	2738
3	11	55	82	198	430	718	1912	3664	5969
4	40	66	88	376	540	801	3458	4725	7012
5	53	73	89	495	628	833	4340	5430	7470
6	60	78	92	553	675	859	4865	5870	7812
7	64	81	94	600	706	874	5245	6191	8032
8	67	81	94	630	729	885	5523	6440	8183
9	69	82	94	654	744	890	5770	6681	8301
10	70	83	94	666	760	896	5888	6877	8400

Table S3: Validation (valid.) and test set RMSE values per Chemprop training iteration for the SurA target: The three replicates with a failed score of +5.0, a failed score of 0.0 and the run where failed compounds were dropped from the training data are shown.

	Failed +5.0									
Iteration	Rep 1		Rep 2		Rep 3		Failed 0.0		Exclude failed	
	valid.	test	valid.	test	valid.	test	valid.	test	valid.	test
2	1.472	1.463	1.458	1.450	1.473	1.443	0.967	0.965	0.778	0.780
3	1.131	1.121	1.131	1.129	1.116	1.123	0.820	0.820	0.710	0.706
4	1.000	1.003	0.999	1.000	0.996	1.000	0.766	0.769	0.686	0.688
5	0.931	0.932	0.932	0.934	0.932	0.928	0.745	0.744	0.681	0.681
6	0.892	0.890	0.888	0.884	0.889	0.888	0.728	0.730	0.677	0.678
7	0.861	0.858	0.854	0.858	0.862	0.862	0.722	0.720	0.674	0.676
8	0.843	0.841	0.833	0.838	0.839	0.842	0.712	0.713	0.672	0.672
9	0.825	0.826	0.821	0.822	0.819	0.821	0.709	0.709	0.671	0.672
10	0.809	0.811	0.810	0.817	0.811	0.814	0.705	0.704	0.670	0.670

Table S4: Validation (valid.) and test set RMSE values per Chemprop training iteration for the GAK target: Results for a failed score of +5.0, a failed score of 0.0 and the three replicate runs where failed compounds were dropped from the training data are shown.

Iteration	Failed +5.0		Failed 0.0		Exclude failed					
					Rep 1		Rep 2		Rep 3	
	valid.	test	valid.	test	valid.	test	valid.	test	valid.	test
2	4.394	4.397	2.344	2.345	1.012	1.010	1.003	1.006	1.009	1.010
3	3.585	3.593	2.011	2.005	0.810	0.813	0.800	0.800	0.801	0.802
4	3.269	3.274	1.907	1.913	0.770	0.774	0.767	0.767	0.765	0.762
5	3.162	3.171	1.899	1.903	0.759	0.761	0.762	0.761	0.755	0.759
6	3.119	3.111	1.898	1.896	0.754	0.756	0.759	0.758	0.756	0.754
7	3.089	3.089	1.894	1.889	0.755	0.756	0.758	0.758	0.755	0.754
8	3.074	3.065	1.900	1.900	0.758	0.758	0.756	0.755	0.755	0.758
9	3.064	3.070	1.907	1.905	0.758	0.758	0.754	0.757	0.756	0.756
10	3.070	3.070	1.912	1.905	0.758	0.758	0.755	0.755	0.756	0.756

Table S5: Recalls for the target SurA with dropped failed compounds when adding 1.56 million compounds, i.e. 0.1% training data per iteration (0.1%), and when adding 156 000 compounds, i.e. 0.01% training data per iteration (0.01%). The shorter runtime was achieved when running 4 Chemprops/GPU, the longer runtime with a single Chemprop/GPU.

	Recall						Runtime d-HH:MM	
	Top 100		Top 1000		Top 10000			
Iteration	0.1%	0.01%	0.1%	0.01%	0.1%	0.01%	0.1%	0.01%
1	0	0	2	0	10	0	22:18 - 1-11:30	06:55 - 20:07
2	64	22	530	170	4868	1183	1-20:36 - 2-23:00	13:49 - 1-16:13
3	81	64	719	452	6609	3561	2-18:54 - 4-10:30	20:44 - 2-12:20
4	91	69	806	521	7408	4433	3-17:12 - 5-22:00	1-03:38 - 3-08:26
5	92	70	839	566	7829	4923	4-15:30 - 7-09:30	1-10:33 - 4-04:33
6	93	72	866	596	8040	5310	5-13:48 - 8-21:00	1-17:28 - 5-00:40
7	93	74	881	618	8265	5579	6-12:06 - 10-08:30	2-00:22 - 5-20:46
8	95	76	890	647	8403	5874	7-10:24 - 11-20:00	2-07:17 - 6-16:53
9	95	77	895	662	8537	6064	8-08:42 - 13-07:30	2-14:11 - 7-12:59
10	96	80	908	675	8657	6222	9-07:00 - 14-19:00	2-21:06 - 8-09:06

Table S6: Recalls and runtime for the target GAK with dropped failed compounds when adding 1.56 million compounds, i.e. 0.1% training data per iteration (0.1%), and when adding 156 000 compounds, i.e. 0.01% training data per iteration (0.01%). The shorter runtime was achieved when running 4 Chemprops/GPU, the longer runtime with a single Chemprop/GPU. The run with the larger training dataset size was terminated after 10 iterations, the run with the smaller training dataset size was continued for a total of 25 iterations.

	Recall						Runtime	
	Top 100		Top 1000		Top 10000		d-HH:MM	
Iteration	0.1%	0.01%	0.1%	0.01%	0.1%	0.01%	0.1%	0.01%
1	0	0	2	0	17	0	21:30 - 34:42	6:50 - 20:02
2	52	4	389	104	2738	656	1-19:00 - 2-21:24	13:40 - 1-16:04
3	82	42	718	330	5969	2285	2-16:30 - 4-08:06	20:29 - 2-12:05
4	88	65	801	491	7012	3402	3-14:00 - 5-18:48	1-03:19 - 3-08:07
5	89	77	833	598	7470	4308	4-11:30 - 7-05:30	1-10:09 - 4-04:09
6	92	81	859	652	7812	4893	5-09:00 - 8-16:12	1-16:59 - 5-00:11
7	94	82	874	679	8032	5304	6-06:30 - 10-02:54	1-23:49 - 5-20:13
8	94	82	885	700	8183	5589	7-04:00 - 11-13:36	2-06:38 - 6-16:14
9	94	83	890	716	8301	5841	8-01:30 - 13-00:18	2-13:28 - 7-12:16
10	94	84	896	725	8400	6045	8-23:00 - 14-11:00	2-20:18 - 8-08:18
11	-	85	-	736	-	6213		3-03:08 - 9-04:20
12	-	85	-	755	-	6436		3-09:58 - 10-00:22
13	-	86	-	771	-	6579		3-16:47 - 10-20:23
14	-	86	-	779	-	6687		3-23:37 - 11-16:25
15	-	86	-	788	-	6821		4-06:27 - 12-12:27
16	-	86	-	793	-	6900		4-13:17 - 13-08:29
17	-	87	-	799	-	6959		4-20:07 - 14-04:31
18	-	87	-	799	-	7016		5-02:56 - 15-00:32
19	-	87	-	803	-	7098		5-09:46 - 15-20:34
20	-	88	-	807	-	7149		5-16:36 - 16-16:36
21	-	88	-	818	-	7230		5-23:26 - 17-12:38
22	-	89	-	824	-	7299		6-06:16 - 18-08:40
23	-	89	-	833	-	7367		6-13:05 - 19-04:41
24	-	90	-	840	-	7435		6-19:55 - 20-00:43
25	-	93	-	850	-	7502		7-02:45 - 20-20:45

Table S7: Recalls of top 100, 1000, and 10000 true virtual hits according to conventional docking obtained in three independent replicates of HASTEN for the target SurA. Failed compounds were assigned a score of +5.0.

	Top 100			Top 1000			Top 10000		
Iteration	Rep 1	Rep 2	Rep 3	Rep 1	Rep 2	Rep 3	Rep 1	Rep 2	Rep 3
1	0	0	0	1	0	1	13	11	12
2	42	41	41	408	402	377	3675	3542	3460
3	70	62	64	630	612	625	5970	5833	5937
4	83	80	77	734	738	721	6877	6877	6826
5	90	88	84	800	789	796	7486	7440	7443
6	93	89	90	836	821	836	7806	7738	7815
7	93	91	92	864	854	859	8045	8019	8056
8	94	92	93	878	872	874	8223	8208	8209
9	94	92	93	885	882	887	8340	8394	8375
10	95	93	94	896	894	899	8477	8500	8500

Table S8: Recalls of top 100, 1000, and 10000 true virtual hits according to conventional docking obtained in three independent replicates of HASTEN for the target GAK. Failed compounds were dropped.

	Top 100			Top 1000			Top 10000		
Iteration	Rep 1	Rep 2	Rep 3	Rep 1	Rep 2	Rep 3	Rep 1	Rep 2	Rep 3
1	0	0	0	2	2	0	17	9	13
2	52	53	64	389	423	477	2738	2774	3224
3	82	82	83	718	733	723	5969	5912	5942
4	88	86	88	801	805	787	7012	7023	6982
5	89	90	92	833	833	825	7470	7513	7479
6	92	90	93	859	854	868	7812	7797	7879
7	94	92	93	874	866	880	8032	8002	8064
8	94	94	93	885	880	891	8183	8156	8207
9	94	94	93	890	887	898	8301	8281	8313
10	94	94	93	896	896	900	8400	8393	8407

Selected Chemprop parameters

Any non-standard parameters or parameters with defaults not being False/None that influence the training procedure (not e.g. output paths or logging) are summarized below:

`-dataset_type`	regression	
`-loss_function`	mse	
`-split_type`	predetermined	*Random split is done with HASTEN*
`-split_sizes`	None	*Split sizes determined by HASTEN as 0.8 (train), 0.1 (test), 0.1 (validation)*
`-num_folds`	1	*Number of folds when performing cross validation*
`-seed`	0	*Chemprop random seed*
`-pytorch_seed`	0	*Pytorch random seed*
`-metric`	rmse	*Metric to use during evaluation and with validation set for early stopping*
`-hidden_size`	300	*Dimensionality of hidden layers in MPN*
`-depth`	3	*Number of message passing steps*
`-dropout`	0.0	*Dropout probability*
`-activation`	ReLU	*Activation function*
`-ffn_num_layers`	2	*Number of layers in FFN after MPN encoding*
`-ensemble_size`	1	*Number of models in ensemble*
`-aggregation`	mean	*Aggregation scheme for atomic vectors into molecular vectors*
`-aggregation_norm`	100	*For norm aggregation, number by which to divide summed up atomic feature*
`-epochs`	30	*Number of epochs to run*
`-warmup_epochs`	2.0	*Number of epochs during which learning rate increases linearly from* `init_lr` *to* `max_lr`
`-init_lr`	0.0001	*Initial learning rate*
`-max_lr`	0.001	*Maximum learning rate*
`-final_lr`	0.0001	*Final learning rate*
`-number_of_molecules`	1	*Number of molecules in each input to the model*
`-num_workers`	8	*Number of workers for the parallel data loading*
`-batch_size`	250	*Batch size*
`-no_cache_mol`	True	*Whether to not cache the RDKit molecule for each SMILES string*

GAK receptor selection and method validation

Re- and cross-docking studies for GAK structure selection

To select the best possible receptor for the GAK target docking study, initially, all available crystallographic complexes of the GAK protein with bound drug-like ligands, as listed in Table **S9**, were considered and prepared as described in the main text. The four crystallographic ligands were likewise prepared with the same routine as described for the ER lead-like library in the main text. We then evaluated all receptor structures in a re-/cross-docking study, using the same docking approach as discussed in the main text. Results are summarized in Table **S9**.

Table S9: Summary of re- and cross-docking results for all GAK structures with bound drug-like ligands. Ligands in bold-face correspond to this structure's native ligand, i.e. the re-docking scenario. For FEF, all reported RMSDs are based on its orientation in PDB-ID 4c57. In the case of IRE, except for receptors 5y7z and 5y80 (both compared with PDB-ID 5y7z), the reported RMSD was achieved in comparison with PDB-ID 5y80. Reported values are heavy atom RMSDs relative to the crystallographic orientation.

**PDB-ID**	**49J**	**824**	**FEF**	**IRE**
**4c57**	8.39	1.55	**1.02**	1.12
**4c58**	4.32	**0.47**	1.87	1.32
**4c59**	0.64	1.31	**1.63**	1.10
**4y8d**	**0.65**	1.05	1.77	3.41
**5y7z**	NA	1.12	1.44	**1.19**
**5y80**	8.34	NA	1.89	**2.02**

Based on those initial results, PDB-ID 5y80 was excluded since the re-docking resulted in an RMSD close to 2 Å and two out of three cross-dockings were unsuccessful when defining docking success by a heavy atom RMSD cutoff of 2Å. We further excluded PDB-ID 4c57, since its native ligand was identical to that of 4c59, but it performed worse in the cross-docking study. We selected one receptor per native crystallographic ligand, namely PDB-IDs 5y7z, 4y8d, 4c58, and 4c59, for further evaluation of their ability to enrich true actives over property-matched decoys.

Generation of a custom GAK actives/decoys dataset

Known actives for the GAK protein were collected from ChEMBL.[1] Any compound with reported activities of at least 1 μM IC_{50}, K_i, or K_d was considered a potential active to mimic a generous selection of potential binders during the virtual screening project. To ensure that the possible enrichment was assessed in the most relevant property space, the retrieved actives were next filtered by their properties to keep only such compounds that fell inside the lead-like criteria of the ERLL library to be used in the screening study.

The remaining 104 lead-like actives were used to generate a set of custom decoys using DUD-E.[2,3] After removal of duplicates from the decoy set, we ended up with a final dataset of 104 actives and 5600 custom decoys, that were prepared as described for the ERLL library in the main text.

Docking performance and enrichment assessment

For the four prioritized GAK receptors (PDB-IDs 5y7z, 4y8d, 4c58, and 4c59), we analyzed the screening performance and enrichment of actives over decoys using custom Python scripts to compute the following metrics: Area under the Receiver Operating Characteristic Curve (ROC, equation **1**), Area under the Accumulation Curve (AUAC, equation **2**), and the Enrichment Factor in the top 1% (EF, equation **3**). The results are reported in Table **S10**.

$$ROC = \frac{1}{(nN)} \sum_{k=2}^{N} F_a(k)[F_i(k) - F_i(k-1)] \tag{1}$$

with n: number of actives in a total of N compounds; $F_a(k)$ and $F_i(k)$: the number of actives and inactives at rank position k, respectively.

$$AUAC = \frac{1}{(2nN)} \sum_{k=0}^{N-1} [F_a(k) + F_a(k+1)] \tag{2}$$

with n: number of actives in a total of N compounds; $F_a(k)$: the number of actives at rank position k.

$$EF = \frac{\frac{a}{n}}{\frac{A}{N}} \tag{3}$$

with a: number of actives in top 1% compounds (n) of a rank-ordered total of N compounds with A actives in total.

Table S10: Screening performance and enrichment obtained with different GAK receptor structures.

PDB-ID	ROC	AUAC	EF
**4y8d**	0.83	0.85	17.29
**4c59**	0.79	0.73	13.21
**5y7z**	0.73	0.75	7.05
**4c58**	0.71	0.73	1.12

Our analysis highlights PDB-ID 4y8d as the most promising screening receptor and demonstrates that it successfully enriches true actives over decoys with the chosen docking protocol Glide HTVS.

References

(1) Mendez, D.; Gaulton, A.; Bento, A. P.; Chambers, J.; De Veij, M.; Félix, E.; Magariños, M. P.; Mosquera, J. F.; Mutowo, P.; Nowotka, M.; Gordillo-Marañón, M.; Hunter, F.; Junco, L.; Mugumbate, G.; Rodriguez-Lopez, M.; Atkinson, F.; Bosc, N.; Radoux, C. J.; Segura-Cabrera, A.; Hersey, A.; Leach, A. R. ChEMBL: towards direct deposition of bioassay data. *Nucleic Acids Res.* **2019**, *47*, D930–D940.

(2) Mysinger, M. M.; Carchia, M.; Irwin, J. J.; Shoichet, B. K. Directory of Useful Decoys, Enhanced (DUD-E): Better Ligands and Decoys for Better Benchmarking. *J. Med. Chem.* **2012**, *55*, 6582–6594.

(3) DUD-E: A Database of Useful Decoys: Enhanced. `https://dude.docking.org/`, [Online; accessed 2023-07-19].
